# Temperature Dependence of the Ring Opening of Cyclopropene Imines on Thorium Metallocenes

**DOI:** 10.1021/acs.inorgchem.3c04213

**Published:** 2024-03-12

**Authors:** Hemanta Deka, Natalia Fridman, Moris S. Eisen

**Affiliations:** † Schulich Faculty of Chemistry, Technion-Israel Institute of Technology, Haifa City 3200003, Israel; ‡ Department of Chemistry, Goalpara College, Goalpara 783101, Assam, India

## Abstract

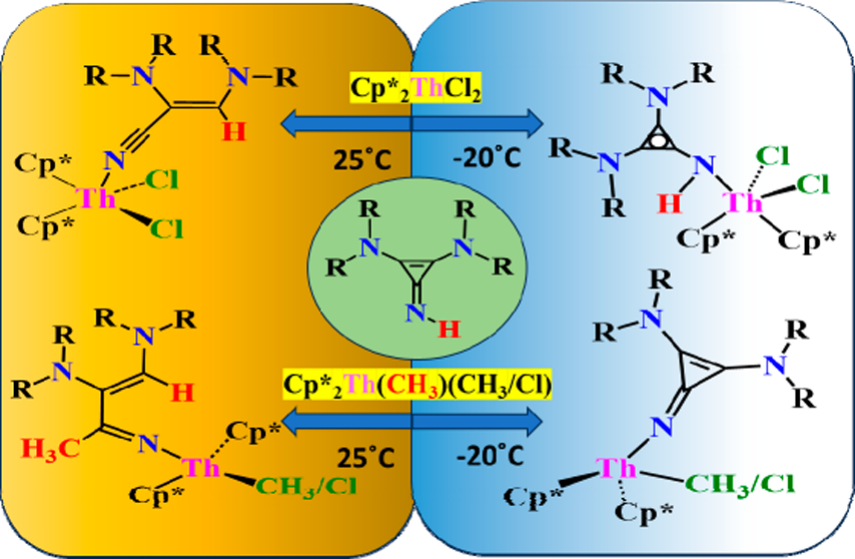

The reactions of two highly strained cyclopropenimine ligands **L1H** and **L2H** (**L1H** = *N*^1^*,N*^1^*,N*^2^*,N*^2^-tetraisopropyl-3-iminocycloprop-1-ene-1,2-diamine, **L2H** = *N*^1^*,N*^1^*,N*^2^*,N*^2^-tetracyclohexyl-3-iminocycloprop-1-ene-1,2-diamine) with three thorium precursors Cp*_2_ThCl_2_, Cp*_2_Th(Cl)(CH_3_), and Cp*_2_Th(CH_3_)_2_ were studied. At −20 °C, **L1H** and **L2H** react with Cp*_2_ThCl_2_ to form **Th1** (**Th1** = Cp*_2_ThCl_2_(L1H)) and **Th2** (**Th2** = Cp*_2_ThCl_2_(L2H)), respectively, where the neutral ligand coordinates to the thorium metal center. Coordination of the ligand to the thorium metal center introduces aromaticity at the cyclopropene ring of the ligand. Reaction at room temperature results in the ring opening of the ligand to form **Th3** (**Th3** = Cp*_2_ThCl_2_((*Z*)-2,3-bis(diisopropylamino)acrylonitrile) and **Th4** (**Th4** = Cp*_2_ThCl_2_((*Z*)-2,3-bis(dicyclohexylamino)acrylonitrile), where the cyclopropenimine converts into a nitrile and coordinates to the thorium metal center. Reaction of **L1H** and **L2H** with Cp*_2_Th(Cl)(CH_3_) and/or Cp*_2_Th(CH_3_)_2_ at −20 °C results in a rapid methanolysis reaction and forms Cp*_2_Th(L1/L2)(CH_3_/Cl)-type complexes **Th5** (**Th5** = Cp*_2_Th(L1)(CH_3_)), **Th6** (**Th6** = Cp*_2_Th(L2)(CH_3_), **Th7** (**Th7** = Cp*_2_Th(L1)(Cl), and **Th8** (**Th8** = Cp*_2_Th(L2)(Cl). On the other hand, at room temperature, these reactions result in a ring opening of the ligand. Room-temperature reaction of **L1H** and **L2H** with Cp*_2_Th(CH_3_)_2_ results in **Th9** (**Th9** = Cp*_2_Th(CH_3_)((*Z*)-3-imino-*N*^1^*,N*^1^*,N*^2^*,N*^2^-tetraisopropylbut-1-ene-1,2-diamine) and **Th10** (**Th10** = Cp*_2_Th(CH_3_)((*Z*)-3-imino-*N*^1^*,N*^1^*,N*^2^*,N*^2^-tetracyclohexylbut-1-ene-1,2-diamine). Similarly, at room temperature, **L1H** and **L2H** react with Cp*_2_Th(Cl)(CH_3_) to form **Th11** (**Th11** = Cp*_2_Th(Cl)((*Z*)-3-imino- *N*^1^*,N*^1^*,N*^2^*,N*^2^-tetraisopropylbut-1-ene-1,2-diamine) and **Th12** (**Th12** = Cp*_2_Th(Cl)((*Z*)-3-imino-*N*^1^*,N*^1^*,N*^2^*,N*^2^-tetracyclohexylbut-1-ene-1,2-diamine). The ring-opening reaction is assisted by the nucleophilic attack of the thorium-coordinated methyl group to the highly strained cyclopropene imine carbon.

## Abstract 2

The reaction of various thorium metallocenes with two cyclopropene imine ligands has been studied at different temperatures. At low temperatures (−20 °C), the cyclopropene imine is coordinated to the metal center, keeping the ring intact. At room temperature, the cyclopropene rings open, forming either a nitrile or an iminato vinyl complex depending on the starting metallocene. Full characterization including the single-crystal structures for all 12 complexes is presented.

## Introduction

**Figure 1:**
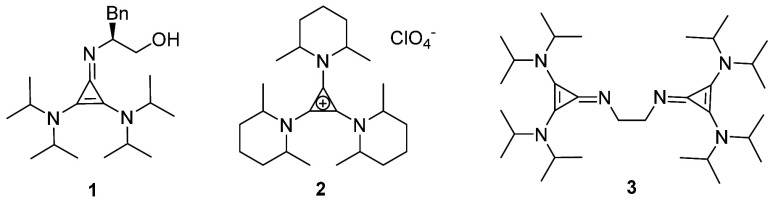
Some recently reported cyclopropenimine-containing compounds.

**Figure 2:**
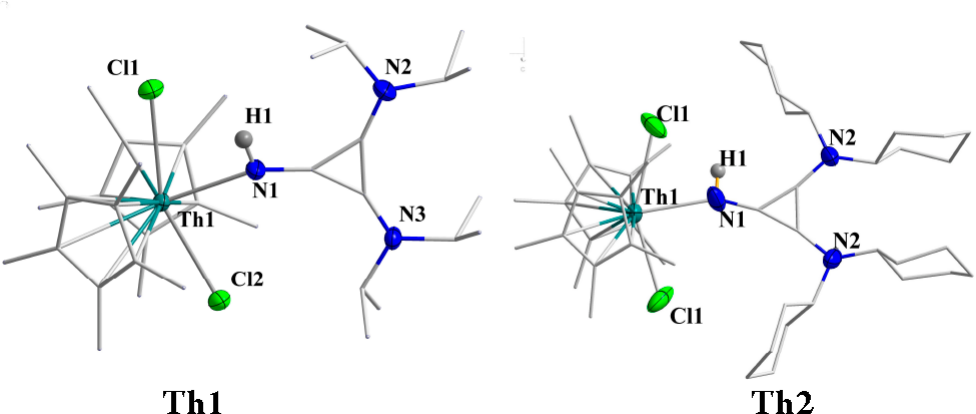
Solid-states structures of **Th1** and **Th2**. H atoms (except imine hydrogen) are omitted for clarity. In the X-ray structure of **Th2**, two disordered structures are obtained, each one with the N1 with 50% occupancy.

**Figure 3:**
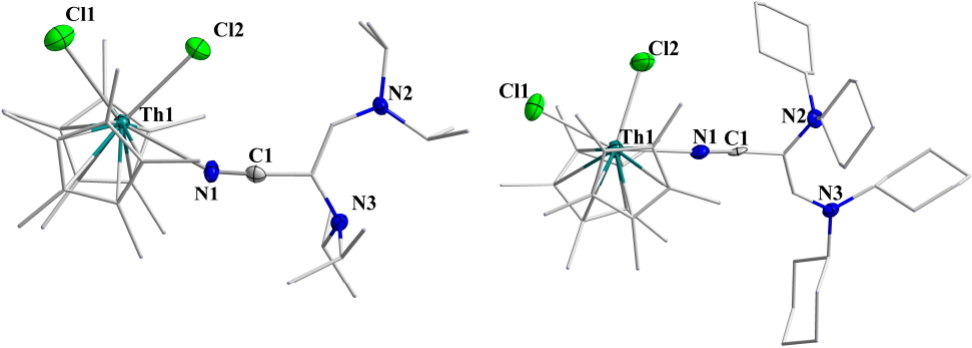
Solid-state structures of complexes **Th3** (left) and **Th4** (right). H atoms are omitted for clarity.

Cyclopropenimines are an important class of organic compounds that have attracted chemists’ attention due to their superbasic performance.^1^ The high basicity of cyclopropenimines arises from the stability of the conjugated acid by forming a Hückel aromatic system.^2^ Among many other superbases, like amidines, guanidines, and phosphazenes, which rely on the combined action of multiple amino substituents, cyclopropenimines have been found to be more appealing because of their application in organic synthesis, including their use in asymmetric organocatalysis.^1,3^ 2,3-Diaminocyclopropenimines with a chiral substitution at the imine-N (Figure 1, **1**) are a new class of organocatalysts which enantioselectively catalyze a wide range of reactions.^4^ Similarly, the trisaminocyclopropenium cation (Figure 1, **2**) was found to be an excellent electrophotocatalyst for various organic transformations like C–H aminations, Michael condensations, etc.^5^ The imine nitrogen on the cyclopropenimine can coordinate to metal centers, creating a new type of N-donor ligand with special properties in their coordination chemistry. Alcarazo and co-workers reported the use of a variety of imine N-substituted bis(diisopropylamino)cyclopropenimines as ligands to form new coordination complexes upon their reactions with {RhCl(CO)_2_}_2_.^6^ Because cyclopropenylidenes serve as good σ-donors and poor π-acceptors, it was envisioned that the imino nitrogen of the cyclopropenimines would serve as a four-electron donor. Hence, the ethylenediamine analog, cyclopropenimine (Figure 1, **3**), indeed was found to allow a 2-fold coordination of Pd(II) to the imine nitrogen atoms.^6,7^

Due to the high ring strain, the cyclopropene ring-containing compounds are prone to undergo ring-opening reactions with nucleophiles, which leads to a new strategy to synthesize various organic compounds that cannot easily be synthesized using classical methods.^8^ In this regard, the transition metal-catalyzed reaction of cyclopropenone with various nucleophiles was broadly studied and well established.^9^ Nanda et al. reported the synthesis of various acrylate and acrylamide derivatives using cyclopropenone as an electrophile with an alcohol or phenol as the nucleophile catalyzed by NHC palladium complexes.^10^ Cyclopropenthione also reacts with amines and alcohols to form the corresponding thioamides and thioesters, respectively, where the reaction was promoted by phosphines.^11^ Marek and co-workers have extensively studied the application of cyclopropene derivatives in various organic syntheses.^12^ However, studies on the ring opening of cyclopropenimine analogs have been somewhat limited.

Our laboratory has focused on expanding the scope of actinide chemistry by developing catalysts based on iminato complexes of thorium and uranium.^13^ The unique characteristics of actinide metals such as their large ionic size, high oxophilicity, and the ability to display high coordination numbers have motivated us to study their coordinative properties and reactivity with various small molecules, which leads to the isolation of new coordination motifs.^14^ As a part of our work in actinide chemistry, here we present the reactivity of substituted cyclopropenimines with various thorium precursor complexes. Different reactivities were observed at different temperatures.

## Results and Discussion

**Scheme 1:**
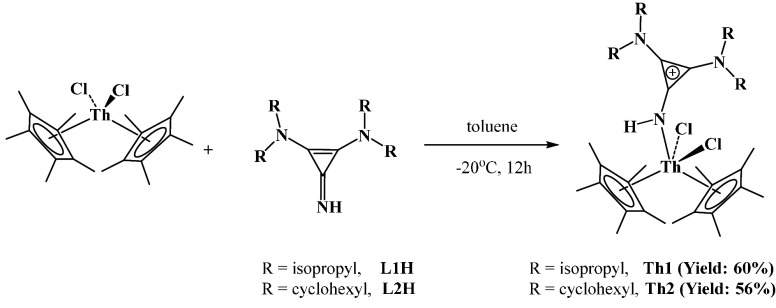
Synthesis of Complexes **Th1** and **Th2**

Hydrochloride salts of the superbasic cyclopropenimine ligands **L1H·HCl** and **L2H·HCl** were synthesized by treating pentachloro cyclopropane with the appropriate amine followed by the addition of ammonia (see the Experimental Section and SI). The resulting hydrochloride salt was neutralized by a NaOH solution to form the crystalline neutral ligands **L1H** and **L2H** in high yields. The neutral **L1H** was first reported by Lambert and co-workers.^1a^ Both ligands contain a superbasic imine moiety with p*K*_a_ = 26.1 (for **L1H**) of the conjugate acid, which is slightly less basic than the previously reported N-heterocyclic iminato ligand 1,3-diisopropyl-1*H*-perimidin-2(3*H*)-imine where the p*K*_a_ of the conjugate acid is 30.4.^15^ Low-temperature (−20 °C) reaction of the neutral ligands, **L1H** and **L2H**, with Cp*_2_ThCl_2_ results in the coordination of the ligands to the metal center to form the complexes **Th1** and **Th2** as white crystalline solids (Scheme 1) with 60% and 56% yields, respectively. Both complexes were characterized using various spectroscopic analysis methods as well as single-crystal X-ray structure determination. The solid-state structure of both complexes is presented in Figure 2. The Th1–N1 bond lengths were found to be 2.454(3) and 2.483(19) Å, respectively, which are much longer compared to the reported imidazoline 2-iminato ligand where the nitrogen is negatively charged and much shorter than the charge-neutral nitrogen atom coordinated to a thorium metal, implying a partial negative charge in the nitrogen atom.^13^ The coordination of the ligand to the metal highly influences its electronic structure. The three C–C bond lengths in the cyclopropenyl ring of **Th1** are quite similar, i.e., 1.387(4), 1.389(4), and 1.390(5) Å, whereas these values are quite different at the free ligand **L1H** (1.412(4), 1.412(4), and 1.365(7) Å). Similarly, for **Th2**, these C–C values at the coordinated ligand are 1.324(3), 1.343(18), and 1.343(18) Å, as compared to their bond lengths at the free ligand **L2H** (1.374 (7), 1.374 (7), 1.350 (7) Å). These results suggest that the cyclopropenium ring gains aromaticity due to its positive charge that is largely present on the aromatic cyclopropenyl ring, represented by the mesomeric structures (Scheme 1). Elongation of the C1–N1 bond length from 1.290(5) to 1.330(5) Å in **Th1** as compared to the same C1–N1 bond in **L1H** and from 1.310(8) to 1.33(2) Å in **Th2** as compared to **L2H** also suggest the accumulation of positive charge at the cyclopropene ring.

Thus, the cyclopropenyl moiety in these molecules can act as an electron reservoir. In the free ligand, it accepts π-electron density from the imine nitrogen, thereby losing some of its aromatic character. Upon coordination, it compensates for the electron density transferred from the nitrogen atom to the metal, recovering aromaticity.

**Scheme 2:**
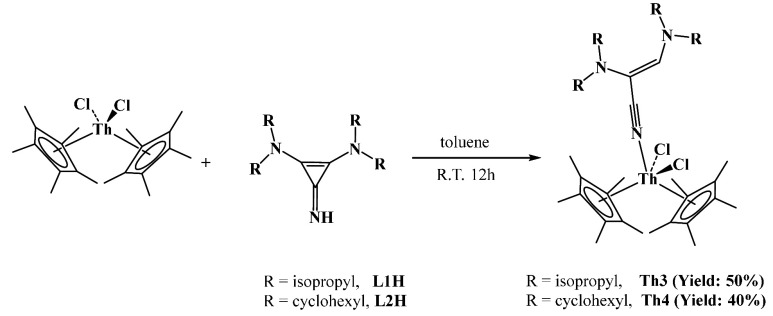
Synthesis of Complexes **Th3** and **Th4**

At room temperature, the Cp*_2_ThCl_2_ and the ligands react differently (Scheme 2). The reaction of an equimolar solution of **L1H** or **L2H** with Cp*_2_ThCl_2_, at room temperature in toluene, forms **Th3** or **Th4** as a white crystalline solid with 50% or 40% yield, respectively. The solid-state structures of **Th3** and **Th4** (Figure 3) revealed the ring opening of the highly stressed cyclopropene rings to form a nitrile ligand, which coordinated to the thorium metal center via the N atom of the nitrile group.

The Th1–N1 bond lengths were found to be 2.606(7) and 2.544(8) Å for **Th3** and **Th4**, respectively, which are even longer than the Th–N1 bond lengths in the starting complexes **Th1** and **Th2**, implying the neutral coordination of the ligand to the metal center. These bond lengths are comparable with the Th–N bond length of the thorium nitrile complex [Cp*_2_Th(NCCH_3_)_5_][BPh_4_]_2_, where the Th–N bond length was found to be in the range of 2.555(2)–2.610(3) Å.^16^

**Scheme 3:**
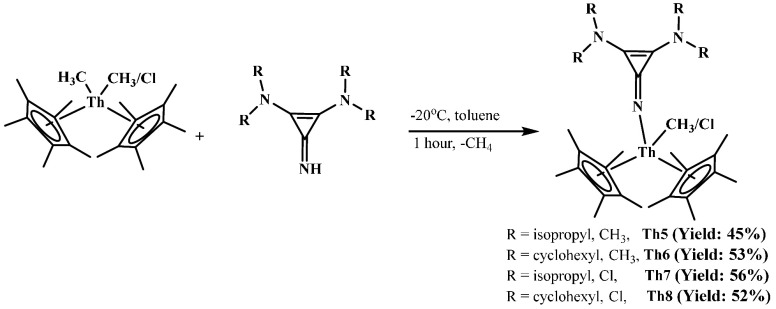
Synthesis of Complexes **Th5**–**Th8**

**Figure 4:**
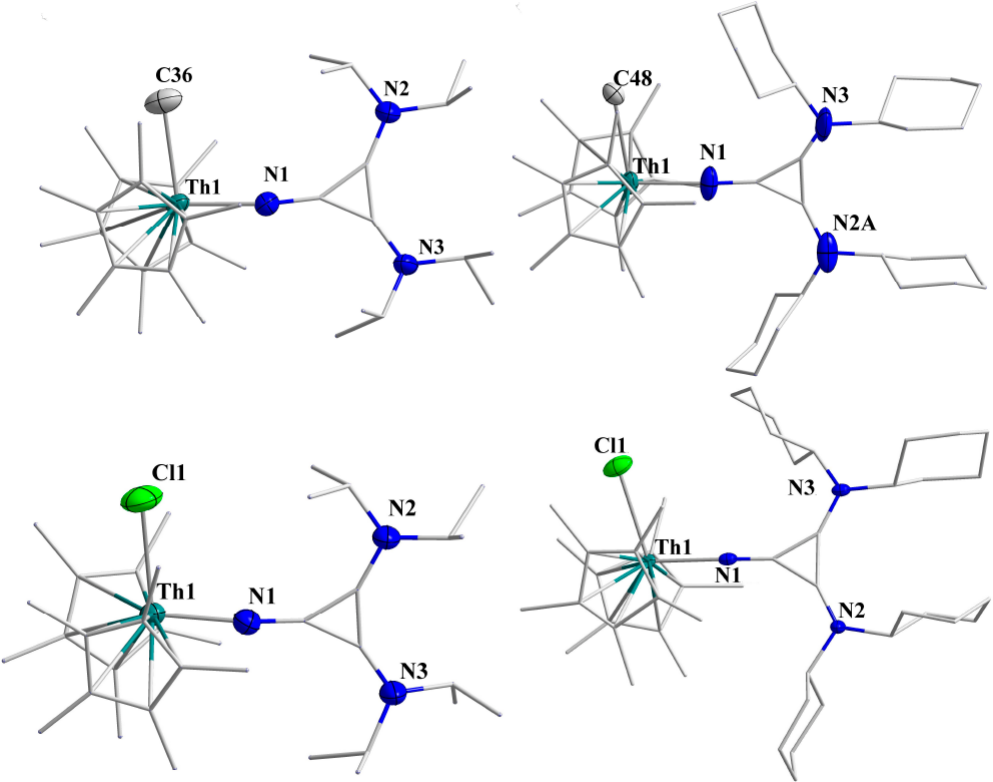
Solid-state structures of **Th5** (top left), **Th6** (top right), **Th7** (bottom left), and **Th8** (bottom right). H atoms are omitted for clarity.

**L1H** and **L2H** react with an equimolar amount of Cp*_2_Th(CH_3_)_2_ and Cp*_2_Th(CH_3_)Cl at −20 °C to quantitatively form complexes **Th5**, **Th6**, **Th7**, and **Th8** with the release of methane gas (Scheme 3). Complexes **Th5–Th8** were obtained as light yellow solids with moderate yield and were fully characterized by spectroscopic measurements. All of the complexes are thermally stable for months at room temperature inside the glovebox. Single crystals of **Th5**, **Th6**, **Th7**, and **Th8** were obtained from a toluene solution after ∼1 week at −20 °C. The solid-state structures are presented in Figure 4. All complexes exhibited distorted tetrahedral coordination around the thorium formed by two Cp* ligands, one methyl or chloro, and one iminato ligand. The Th–CH_3_ bond lengths are 2.502(7) and 2.541(9) Å for **Th5** and **Th6**, respectively. The methyl group shows upfield signals at 0.27 and 0.29 ppm in the ^1^H NMR spectrum, indicating the coordination to the metal center. The Th–Cl bond lengths are 2.6788(16) Å for **Th7** and 2.711(8) Å for **Th8**, which are ∼0.1 Å longer than those in the complex Cp*_2_ThCl_2_.^17^ The corresponding bond length for Cp*_2_ThCl_2_ was 2.601 Å. The C–C bond lengths adjacent to the imine are 1.398 Å on average, which is longer than the distal C–C bond with a bond length of 1.353 Å on average, which indicates electron localization forming the double bonds (due to disorder in the structure of complex **Th8**, the corresponding bond lengths are excluded while taking average bond lengths). The C–N imine bond lengths are 1.306(8), 1.297(6), 1.296(8), and 1.306(3) Å for complexes **Th5**, **Th6**, **Th7**, and **Th8**, respectively, which are shorter than those in complexes **Th1** and **Th2**.

This shortening at the C–N imine bonds indicates that the electron density is localized over the C–N imine bond and one C–C bond distal to the imine carbon. The Th–N1_imine_ bond distances of 2.175(6), 2.150(4), 2.135(6), and 2.100(2) Å for **Th5**, **Th6**, **Th7**, and **Th8**, respectively, are similar to the distances found in the reported analogous iminato complexes in the range from ∼2.1 to 2.3 Å.^13^ This implies that complexes **Th5**–**Th8** are formed by abstracting the acidic imine proton of the ligand by the methyl group coordinated to the thorium, releasing methane gas as reported for other imidazoline iminato complexes.

**Scheme 4:**
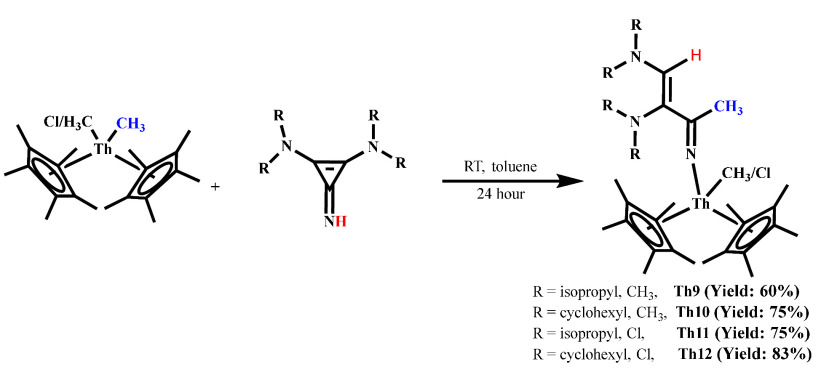
Synthesis of Complexes **Th9–Th12**

**Figure 5:**
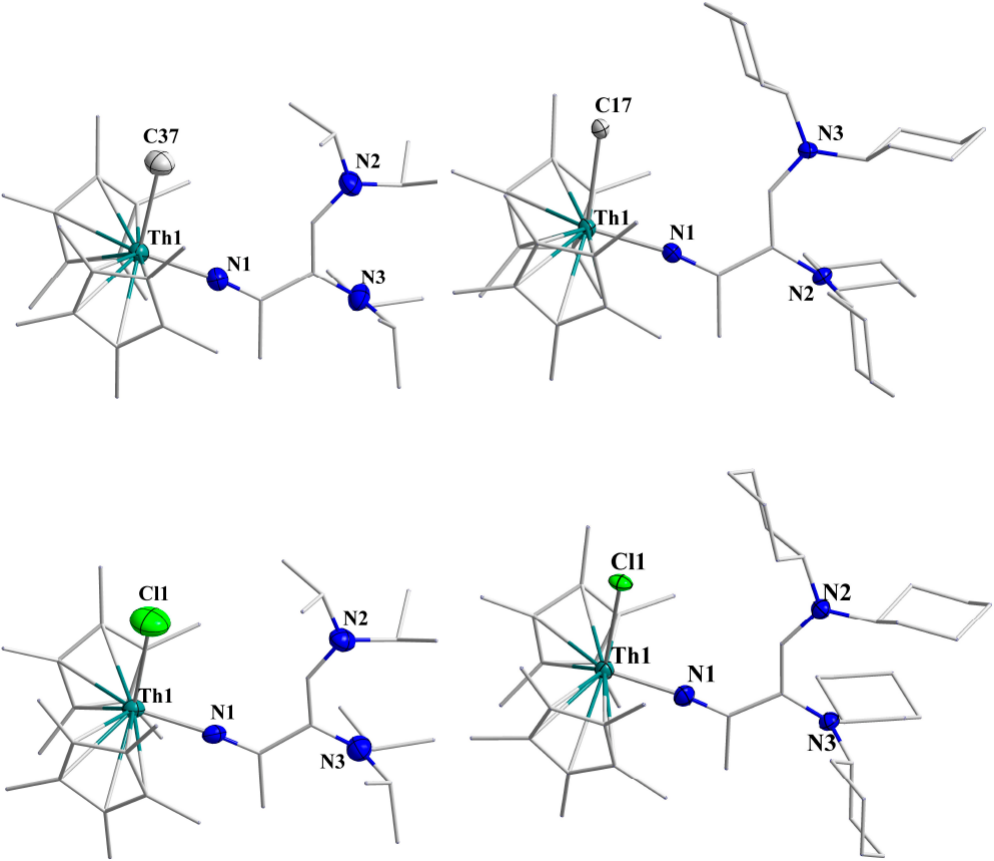
Solid-state structures of **Th9** (top left), **Th10** (top right), **Th11** (bottom left), and **Th12** (bottom right). H atoms are omitted for clarity.

At room temperature, the metal precursors and the ligands react in a different manner. Since **L1H** and **L2H** are superbases, there is a competition between abstracting the imine hydrogen by the coordinated methyl group, releasing methane, or a nucleophilic attack by the methyl group to the active imine carbon opening the cyclopropene ring. At low temperatures, the first reaction was predominant to form complexes **Th5**–**Th8**, where the coordinated methyl group dissociates as methane and the cyclopropene ring remains intact. At room temperature, the reaction of **L1H** and **L2H** with complexes Cp*_2_Th(CH_3_)_2_ and/or Cp*_2_Th(CH_3_)Cl forms complexes **Th9**–**Th12**, respectively, with good yields (Scheme 4). The singlet signal around 7.37 ppm in the ^1^H NMR for complexes **Th9**–**Th12** indicates the formation of a vinyl hydrogen, which is formed via a proton transfer from the imine nitrogen to the alkene carbon. The proton NMR signals of the coordinated methyl group appear between 0.15 and 0.16 ppm for complexes **Th9** and **Th10**, respectively. This area was clear in the case of complexes **Th11** and **Th12**. Formation of **Th5** was also observed even at room temperature with the formation of **Th9** with almost a 30% yield of **Th5**. Complexes **Th9**–**Th12** were structurally characterized, and the solid-state structures are shown in Figure 5. In complexes **Th9**–**Th12**, the ligands **L1H** and **L2H** have been modified via nucleophilic addition of the coordinated methyl group to open the strained cyclopropene ring to form an enimine ligand, which coordinated to the thorium metal via the iminato nitrogen. The Th–N1 bond distances of 2.187(4), 2.181(3), 2.180(6) and 2.164(7) Å are relatively longer as compared to those of complexes **Th5**–**Th8**, where the cyclopropene ring was intact. The Th–CH_3_ bond lengths of 2.521(4) and 2.607(4) Å for **Th9** and **Th10**, respectively, are also longer as compared to those of complexes **Th5** and **Th6.**

The Th–Cl bond lengths are 2.682(3) and 2.825(2) Å for complexes **Th11** and **Th12**, respectively, which are also slightly longer than those for **Th7** and **Th8**.

Complexes **Th9**–**Th12** are formed by the nucleophilic attack of the coordinated methyl group to the active imine group of the strained cyclopropene imine. This results in the cyclopropene ring opening and the formation of a new C–CH_3_ bond. This methyl group shows ^1^H NMR signals at 2.08, 2.28, 2.10, and 2.17 ppm for complexes **Th9**, **Th10**, **Th11**, and **Th12**, respectively. The imine hydrogen migrates to the ring carbon, forming a vinylic hydrogen, which was observed in the ^1^H NMR between 7.37 and 7.53 ppm for complexes **Th9**–**Th12**. The imine C–N bond lengths are 1.273(6), 1.286(5), 1.276(9), and 1.286(10) Å for complexes **Th9**, **Th10**, **Th11**, and **Th12**, respectively, which are shorter than those at the ring closed ligands, indicating no charge delocalization and the pure double-bond character of the iminato ligand.

**Scheme 5:**
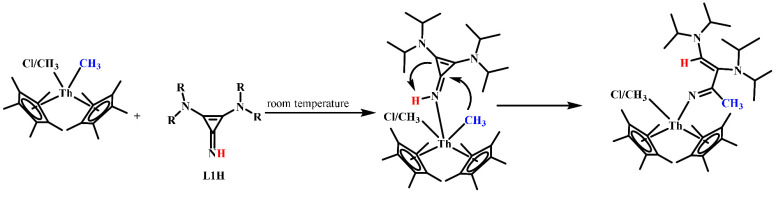
Mechanism of Cyclopropenimine Ring Opening

A plausible mechanism for the ring opening of cyclopropenimine was investigated following ^1^H NMR studies of the reaction of Cp*_2_Th(CH_3_)_2_ and **L1H** (Scheme 5). At room temperature, an immediate reaction of the complex Cp*_2_Th(CH_3_)_2_ and **L1H** shows a signal at 6.35 ppm assigned to the NH peak of the coordinated ligand to Cp*_2_Th(CH_3_)_2_ (see the Supporting Information).

This signal gradually decreases and finally disappears, which indicates the consumption of the coordinated **L1H**. Simultaneously, a new signal at 7.37 ppm gradually appears, assigned to the vinylic hydrogen of the ring-opened iminato ligand. At low temperatures, the thorium-coordinated methyl group abstracts the NH proton of the coordinated ligand to form complex **Th5**.

## Conclusions

This contribution describes the synthesis, characterization, and thermal stability of actinide complexes containing cyclopropene imine as ligands. The formation of the respective complexes highly depends on the reaction temperature. Studies using cyclopropene imines as ligands are very rare and this is the first example of preparing cyclopropene iminato complexes. At −20 °C, ligands **L1H** and **L2H** react with three different thorium metallocenes to form complexes **Th1**, **Th2**, **Th5**, **Th6**, **Th7**, and **Th8**, where the cyclopropene rings remain intact. Remarkably, an unprecedented ring-opening of the cyclopropene to a linear imine or nitrile was observed when the reaction was carried out at room temperature.

## Experimental Section

### General Considerations

All experiments containing air-sensitive materials were performed inside a nitrogen-filled Innovative Technologies glovebox with a medium-capacity recirculator (1–2 ppm of O_2_) or with flame-dried rigorous exclusion of oxygen and moisture using standard Schlenk technique or J-Young Teflon valve-sealed NMR tubes on a dual-manifold Schlenk line interfaced to a high-vacuum (10^–5^ Torr) line. Argon and nitrogen were purified by passage through a MnO oxygen-removal column and a Davison 4 Å molecular sieve column. Analytically pure solvents were distilled under vacuum from Na/K alloy, and air was removed by the freeze–pump– thaw technique (benzene-*d*_6_ (Cambridge Isotopes), toluene (Bio-Lab), and diethyl ether (Bio-Lab)). The thorium precursors Cp*_2_Th(Me)_2_, Cp*_2_ThCl_2,_ and Cp*_2_Th(Me)(Cl) were prepared following published procedures.^17^**L1H** was prepared by following a reported procedure (see the Supporting Information).^1a^ All of the aforementioned reagents were stored in an inert atmosphere glovebox before use. NMR spectra were recorded on either a Bruker Avance 300 or a Bruker Avance III 400 spectrometer. Chemical shifts for ^1^H and ^13^C NMR are referenced to internal protio solvent and reported relative to tetramethylsilane. *J* values are reported for ^1^H NMR coupling constants in hertz (Hz). The single-crystal material was immersed in perfluoropolyalkyl ether, quickly fished out with a glass rod, and mounted on an Apex II Bruker diffractometer under a cold stream of nitrogen at 200 K. Data collection was performed using monochromated Mo Kα radiation using φ and ω scans to cover the Ewald sphere.^18^ Accurate cell parameters were obtained with the amount of indicated reflections.^19^ The structure was solved by SHELXS-97 direct methods and refined by the SHELXL97 program package.^20^ The atoms were refined anisotropically. Hydrogen atoms were included using the riding model. Figures were drawn (50% probability thermal ellipsoids) using Diamond V3.1.8

#### Preparation of 3-Imino-bis(dicyclohexylamino)cycloprop-1-ene Hydrochloride **(L2H·HCl**)

An ammonia solution (15 mL) was slowly added to a solution of 1-chloro-2,3-bis(dicyclohexylamino)cycloprop-2-en-1-ylium chloride^1a,3a^ (Supporting Information) (4.32 g) in dichloromethane (50 mL) at 0 °C. The resulting solution was stirred at 0 °C for 30 min. The organic part was separated, and the aqueous part was washed with 50 mL of DCM. Each organic part was combined, dried, and washed with ethyl acetate to obtain **L2H·HCl** (3 g, 67% yield). Anal. Calcd for C_27_H_46_ClN_3_: C, 72.37; H, 10.35; N, 9.38. Found: C, 71.21; H, 10.12; N, 9.45. ^1^H NMR (CDCl_3_, 300 MHz, 298 K): (δ_ppm_) 6.17 (s, 1H, N*H*), 3.14 (m, 4H, C*H*), 1.76–1.20 (m, 40H, cyclohexyl). ^13^C NMR (CDCl_3_, 75 MHz, 298 K): (δ_ppm_) 115.1, 113.9, 32.9, 25.7, 24.7.

#### Preparation of 3-Imino-bis(dicyclohexylamino)cycloprop-1-ene (**L2H**)

A 447 mg (1 mmol) amount of **L2H·HCl** was dissolved in 25 mL of dichloromethane, and 5 mL of a 1 M aqueous NaOH solution was added. The mixture was stirred vigorously at room temperature. The organic part was separated, and the aqueous part was washed with 25 mL of DCM. Each organic part was combined, dried, and washed with ethyl acetate to obtain **L2H** (350 mg, 85% yield). Anal. Calcd for C_27_H_46_ClN_3_: C, 78.77; H, 11.02; N, 10.21. Found: C, 77.71; H, 11.22; N, 10.11. ^1^H NMR (C_6_D_6_, 300 MHz, 298 K): (δ_ppm_) 4.71 (s, 1H, N*H*), 3.00 (p, 4H, *J* = 6.6 Hz, (CH_3_)_2_C*H*), 1.69 (s, 24H, cyclohexyl), 1.51 (s, 4H, cyclohexyl), 1.17 (s, 12H, cyclohexyl). ^13^C NMR (CDCl_3_, 75 MHz, 298 K): (δ_ppm_) 122.8, 120.8 58.9, 33.1, 25.9, 24.9, 22.9. Mass (APCI, *m*/*z*): calcd (M + 1) 412.3692, obsd 412.3750 Da.

#### Preparation of Cp*_2_Th(L1H)Cl_2_ (**Th1**)

Inside a glovebox, 57 mg (0.1 mmol) of Cp*_2_ThCl_2_ was dissolved in 3 mL of toluene in a glass vial equipped with a magnetic stir bar. At the same time, 25 mg (0.1 mmol) of **L1H** was dissolved in 1 mL of toluene in a separate glass vial. Each solution was allowed to cool inside a refrigerator at −20 °C for 1 h. Then, the vials were taken out of the refrigerator, and the solution of **L1H** was added to the solution of Cp*_2_ThCl_2_ as quickly as possible. The reaction mixture was stirred for 1 min and then placed inside the refrigerator. Colorless crystals of **Th1** were obtained after 1 week. Yield: 50 mg, 60%. ^1^H NMR (C_6_D_6_, 300 MHz, 298 K): (δ_ppm_) 6.16 (s, 1H, N*H*), 3.81 (p, 4H, *J* = 6 Hz, C*H*), 2.33 (s, 30H, C_5_(C*H*_3_)_5_), 1.03 (d, *J* = 6 Hz, 24H, *CH*_*3*_). ^13^C NMR (C_6_D_6_, 75 MHz, 298 K): (δ_ppm_) (3-membered ring carbon was not observed) 125.0 (*C*_5_(CH_3_)_5_), 49.3 (*C*H), 22.3 (*C*H_3_), 12.8 (C_5_(*C*H_3_)_5_). Mass (APCI, *m*/*z*): 788.4802 Da (M – Cl).

#### Preparation of Cp*_2_Th(L2H)Cl_2_ (**Th2**)

**Th2** was prepared applying a similar procedure to that for **Th1** using 57 mg of Cp*_2_ThCl_2_ and 41 mg of **L2H.** Yield: 55 mg, 56%. ^1^H NMR (C_6_D_6_, 300 MHz, 298 K): (δ_ppm_) 6.15 (s, 1H, N*H*), 3.58 (m, 4H, C*H*), 2.38 (s, 30H, C_5_(C*H*_3_)_5_), 1.74 (m, 40H, cyclohexyl). ^13^C NMR (C_6_D_6_, 75 MHz, 298 K): (δ_ppm_) 137.9 (cyclopropene ring carbon), 129.3 (cyclopropene ring carbon), 125.0 (*C*_5_(CH_3_)_5_), 58.2 (*C*H), 33.2 (cyclohexyl), 25.9 (cyclohexyl), (25.2 (cyclohexyl), 12.9 (C_5_(*C*H_3_)_5_). Mass (APCI, *m*/*z*): 948.6090 Da (M – Cl).

#### Preparation of Cp*_2_Th((*Z*)-2,3-Bis(diisopropylamino)acrylonitrile)Cl_2_ (**Th3**)

To a toluene solution containing 57 mg (0.1 mmol) of Cp*_2_ThCl_2_ was added a toluene solution containing 25 mg (0.1 mmol) of **L1H**. The reaction mixture was stirred for 24 h at room temperature. The reaction mixture was then dried in a vacuum, and the crude product was recrystallized in a THF–hexane mixture to obtain colorless **Th3**. Yield: 40 mg, 50%. ^1^H NMR (C_6_D_6_, 300 MHz, 298 K): (δ_ppm_) 6.92 (s, 1H, C*H*), 3.22 (p, 4H, *J* = 6 Hz, C*H*), 2.31 (s, 30H, C_5_(C*H*_3_)_5_), 1.17–1.07 (m, 12H, *CH*_*3*_), 0.81 (s, 1H, *CH*_*3*_). ^13^C NMR (C_6_D_6_, 75 MHz, 298 K): (δ_ppm_) 129.3 (C), 128.6 (*C*H), 126.1 (*C*_5_(CH_3_)_5_), 125.7 (CN), 49.75 (*C*H), 23.1, 22.0, 21.4, 20.1 (*C*H_3_), 12.6 (C_5_(*C*H_3_)_5_). Due to weak coordination of the nitrile ligand, a molecular ion peak was not observed.

#### Preparation of Cp*_2_Th((*Z*)-2,3-Bis(dicyclohexylamino)acrylonitrile)Cl_2_ (**Th4**)

**Th4** was prepared applying a similar procedure to that for **Th3** using 57 mg of Cp*_2_ThCl_2_ and 41 mg of **L2H**. Yield: 40 mg, 40%. ^1^H NMR (C_6_D_6_, 300 MHz, 298 K): (δ_ppm_) 7.71 (s, 1H, C*H*), 2.87 (m, 4H, cyclohexyl C*H*), 2.53 (s, 30H, C_5_(C*H*_3_)_5_), 1.73–1.07 (m, 40H, cyclohexyl). ^13^C NMR (C_6_D_6_, 75 MHz, 298 K): (δ_ppm_) 129.3 (*C*H), 125.0 (*C*_5_(CH_3_)_5_), 121.5 (*C*N), 113.9 (*C*CN), 58.2 (*C*H), 33.1 (*C*H_2_), 26.3 (*C*H_2_), 25.0 (*C*H_2_), 13.2 (C_5_(*C*H_3_)_5_). Due to weak coordination of the nitrile ligand, a molecular ion peak was not observed.

#### Preparation of Cp*_2_Th(L1)(CH_3_) (**Th5**)

Inside a glovebox, 53 mg (0.1 mmol) of Cp*_2_Th(CH_3_)_2_ was dissolved in 3 mL of toluene in a glass vial equipped with a magnetic stir bar. At the same time, 25 mg (0.1 mmol) of **L1H** was dissolved in 1 mL of toluene in a separate glass vial. Each solution was allowed to cool inside a refrigerator at −20 °C for 1 h. Then, the vials were taken out of the refrigerator, and the solution of **L1H** was added to the solution of Cp*_2_Th(CH_3_)_2_ as quickly as possible. The reaction mixture was stirred for 1 h and then placed inside the refrigerator. Light-yellow crystals of **Th5** were obtained after 1 week. Yield: 35 mg, 45%. ^1^H NMR (C_6_D_6_, 300 MHz, 298 K): (δ_ppm_) 3.28 (p, *J* = 7 Hz, 4H, C*H*), 2.22 (s, 30H, C_5_(C*H*_3_)_5_), 1.13 (d, *J* = 7 Hz, 24H, *CH*_*3*_), 0.37 (s, 3H, Th–C*H*_3_). ^13^C NMR (C_6_D_6_, 75 MHz, 298 K): (δ_ppm_) (3-membered ring carbon was not observed) 120.5 (*C*_5_(CH_3_)_5_), 50.6 (Th–*C*H_3_), 49.5 (*C*H), 22.8 (*C*H_3_), 11.7 (C_5_(*C*H_3_)_5_). Mass (APCI, *m*/*z*): 767.6708 Da.

#### Preparation of Cp*_2_Th(L2)(CH_3_) (**Th6**)

**Th6** was prepared applying a similar procedure to that for **Th5** using 53 mg of Cp*Th(CH_3_)_2_ and 41 mg of **L2H**. Yield: 50 mg, 53%. ^1^H NMR (C_6_D_6_, 300 MHz, 298 K): (δ_ppm_) 3.06 (m, 4H, cyclohexyl C*H*), 2.24 (s, 30H, C_5_(C*H*_3_)_5_), 1.82–1.29 (m, 40H, cyclohexyl), 0.29 (s, 3 HTh–C*H*_3_). ^13^C NMR (C_6_D_6_, 75 MHz, 298 K): (δ_ppm_) 123.0 (cyclopropene ring carbon), 120.4 (*C*_5_(CH_3_)_5_), 58.5 (*C*H), 52.1 (Th-*C*H_3_), 33.4 (cyclohexyl), 26.6 (cyclohexyl), 25.4 (cyclohexyl), 11.7 (C_5_(*C*H_3_)_5_). Mass (APCI, *m*/*z*): 928.6044 Da.

#### Preparation of Cp*_2_Th(L1)(Cl) (**Th7**)

**Th7** was prepared applying a similar procedure to that for **Th5** using 55 mg of Cp*_2_Th(CH_3_)(Cl) and 25 mg of **L1H**. Yield: 45 mg, 56%. ^1^H NMR (toluene-*d*_8_, 300 MHz, 298 K): (δ_ppm_) 3.32 (*p*, *J* = 7 Hz, 4H, C*H*), 2.22 (s, 30H, C_5_(C*H*_3_)_5_), 1.11 (d, *J* = 7 Hz, 24H, *CH*_*3*_). ^13^C NMR (toluene-*d*_8_, 75 MHz, 298 K): (δ_ppm_) (3-membered ring carbon was not observed) 123.02 (*C*_5_(CH_3_)_5_), 49.6 (*C*H), 22.7 (*C*H_3_), 11.8 (C_5_(*C*H_3_)_5_). Mass (APCI, *m*/*z*): 787.6799 Da.

#### Preparation of Cp*_2_Th(L2)(Cl) (**Th8**)

**Th8** was prepared applying a similar procedure to that for **Th5** using 55 mg of Cp*_2_Th(CH_3_)(Cl) and 41 mg of **L2H**. Yield: 50 mg, 52%. ^1^H NMR (C_6_D_6_, 300 MHz, 298 K): (δ_ppm_) 3.12 (b, 4H, cyclohexyl C*H*), 2.30 (s, 30H, C_5_(C*H*_3_)_5_), 1.86–1.25 (m, 40H, cyclohexyl), 0.29 (s, 3 HTh–C*H*_3_). ^13^C NMR (C_6_D_6_, 75 MHz, 298 K): (δ_ppm_) (3-membered ring carbon was not observed) 125.0 (*C*_5_(CH_3_)_5_), 58.2 (*C*H), 33.2 (cyclohexyl), 26.4(cyclohexyl), 25.9 (cyclohexyl), 12.8 (C_5_(*C*H_3_)_5_). Mass (APCI, *m*/*z*): 948.6124 Da.

#### Preparation of Cp*_2_Th(CH_3_)((*Z*)-2,3-Bis(diisopropylamino)acrylonitrile) (**Th9**)

To a toluene solution containing 53 mg (0.1 mmol) of Cp*_2_Th(CH_3_)_2_ was added a toluene solution containing 25 mg (0.1 mmol) of **L1H**. The reaction mixture was stirred for 24 h at room temperature. The reaction mixture was then dried in vacuum, and the crude product was recrystallized in a diethyl ether–hexane mixture to obtain light yellow **Th9**. Yield: 70 mg, 60%. ^1^H NMR (with a 30% mixture of **Th5**, C_6_D_6_, 300 MHz, 298 K): (δ_ppm_) 7.37 (s, 1H, C*H*), 3.46 (p, 2H, *J* = 6 Hz, C*H*), 3.30 (p, 2H, *J* = 6 Hz, C*H*), 2.10 (s, 30H, C_5_(C*H*_3_)_5_), 2.08 (s, 3H, C*H*_3_), 1.16 (d, 12H, *J* = 6 Hz, C*H*_*3*_), 1.14 (d, 12H, *J* = 6 Hz, C*H*_*3*_) 0.15 (s, 3H, Th–*CH*_*3*_). ^13^C NMR (C_6_D_6_, 75 MHz, 298 K): (δ_ppm_) 179.9 (*C*=N), 137.1 (*C*=C), 121.1 (*C*_5_(CH_3_)_5_), 116.9 (C=*C*), 68.0 (*C*H), 58.8 (Th–*C*H_3_), 53.1 (*C*H), 45.3 (N=C(*C*H_3_)), 32.3 (*C*H_3_), 22.8 (*C*H_3_), 11.3 (C_5_(*C*H_3_)_5_). Mass (APCI, *m*/*z*): 784.5256 Da.

#### Preparation of Cp*_2_Th(CH_3_)((*Z*)-2,3-Bis(dicyclohexylamino)acrylonitrile) (**Th10**)

**Th10** was prepared applying a similar procedure to that for **Th9** using 53 mg of Cp*_2_Th(CH_3_)_2_ and 41 mg of **L2H**. Yield: 70 mg, 75%. ^1^H NMR (C_6_D_6_, 300 MHz, 298 K): (δ_ppm_) 7.38 (s, 1H, C*H*), 3.14 (m, 4H, cyclohexyl C*H*), 2.13 (s, 30H, C_5_(C*H*_3_)_5_), 2.21 (s, 3H, N=C–C*H*_3_), 1.80–1.23 (m, 40H, cyclohexyl), 0.17 (s, 3H, Th–C*H*_3_). ^13^C NMR (C_6_D_6_, 75 MHz, 298 K): (δ_ppm_) 180.6 (*C*=N), 137.0 (*C*=C), 121.1 (*C*_5_(CH_3_)_5_), 116.0 (C=*C*), 58.7 (*C*H), 58.0 (*C*H), 33.4 (cyclohexyl), 32.6 (cyclohexyl), 27.2 (cyclohexyl), 26.6 (cyclohexyl), 26.6 (cyclohexyl), 26.3 (cyclohexyl), 11.3 (C_5_(*C*H_3_)_5_). Mass (APCI, *m*/*z*): 945.8043 Da.

#### Preparation of Cp*_2_Th(Cl)((*Z*)-2,3-Bis(diisopropylamino)acrylonitrile) (**Th11**)

**Th11** was prepared applying a similar procedure to that for **Th9** using 55 mg of Cp*_2_Th(CH_3_)(Cl) and 25 mg of **L1H**. Yield: 60 mg, 75%. ^1^H NMR (C_6_D_6_, 300 MHz, 298 K): (δ_ppm_) 7.53 (s, 1H, C*H*), 3.44 (p, 2H, *J* = 6 Hz, C*H*), 3.24 (p, 2H, *J* = 6 Hz, C*H*), 2.16 (s, 30H, C_5_(C*H*_3_)_5_), 2.10 (s, 3H, C*H*_3_), 1.14 (d, 12H, *J* = 6 Hz, C*H*_*3*_). ^13^C NMR (C_6_D_6_, 75 MHz, 298 K): (δ_ppm_) 182.2 (*C*=N), 138.9 (*C*=C), 123.7 (*C*_5_(CH_3_)_5_), 121.1 (C=*C*), 49.7 (*C*H), 49.3 (*C*H), 45.7 (N=C(*C*H_3_)), 22.8 (*C*H_3_), 22.2 (*C*H_3_), 11.5 (C_5_(*C*H_3_)_5_). Mass (APCI, *m*/*z*): 804.7542 Da.

#### Preparation of Cp*_2_Th(Cl)((*Z*)-2,3-Bis(dicyclohexylamino)acrylonitrile) (**Th12**)

**Th12** was prepared applying a similar procedure to that for **Th9** using 55 mg of Cp*_2_Th(CH_3_)(Cl) and 41 mg of **L2H**. Yield: 80 mg, 83%. ^1^H NMR (C_6_D_6_, 300 MHz, 298 K): (δ_ppm_) 7.37 (s, 1H, C*H*), 3.11 (m, 4H, cyclohexyl C*H*), 2.17 (s, 3H, N=C–C*H*_3_), 2.12 (s, 30H, C_5_(C*H*_3_)_5_), 1.81–1.30 (m, 40H, cyclohexyl), 0.16 (s, 3H, Th–C*H*_3_). Due to the very low solubility, ^13^C NMR measurements were not taken. Mass (APCI, *m*/*z*): 964.8982 Da.

## Supporting Information Available

Experimental details, characterization data, kinetics, data, labeling experiments, crystallographic data, and pertinent refinement parameters (PDF)
