# Supplementary material for: Temperature Dependence of the Ring Opening of Cyclopropene Imines on Thorium Metallocenes
Source: Inorg Chem. 2024 Mar 12;63(21):9572–8. doi: 10.1021/acs.inorgchem.3c04213 (PMC11134510; doi:10.1021/acs.inorgchem.3c04213)
Supplement: Supplementary file 1 — ic3c04213_si_001.pdf [file ic3c04213_si_001.pdf]

Supporting Information

Temperature Dependence of the Ring-Opening of Cyclopropene Imines on Thorium Metallocenes

Hemanta Deka,[a,b] Natalia Fridman[a] and Moris S. Eisen[a]*

[a]Schulich Faculty of Chemistry, Technion-Israel Institute of Technology, Haifa City 3200003, Israel

[b]Department of Chemistry, Goalpara College, Goalpara-783101, Assam, India

E-mail: chmoris@technion.ac.il

Fax = Tel: + 972-4-8292680

Table of Contents

1. Schematic representation for the ligands syntheses S2

2. Synthesis of the neutral ligands S3

Table S1 and Table S2: Crystallographic tables of all complexes S4

X-ray structure, NMR, and mass spectra of ligands and complexes S6

3. References S31

1. Schematic representation for the ligand synthesis

Scheme S1. Synthesis of ligands.

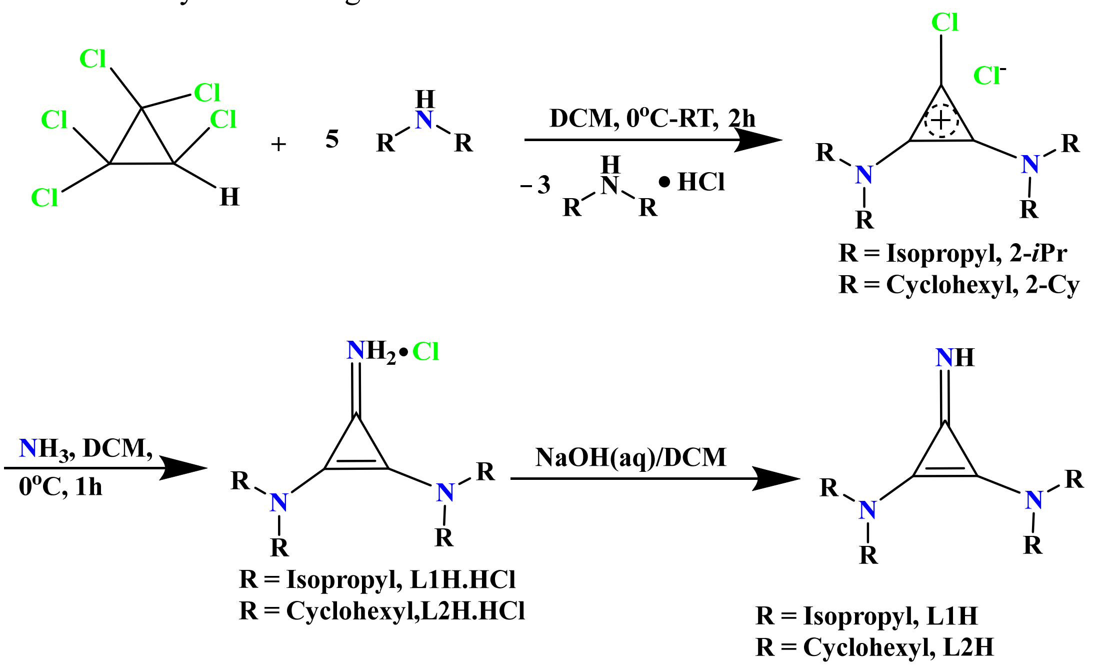

2. Synthesis of the neutral ligands

2.1. 1-chloro-2,3-bis(diisopropylamino)cycloprop-2-en-1-ylium chloride **(2-*i*Pr)**

Diisopropylamine (8.4 mL, 60 mmol, 6.0 equiv) was slowly added to a solution of pentachlorocyclpropane (1.3 ml, 10 mmol, 1.0 equiv) in dichloromethane (100 mL) at 0°C. The resulting solution was stirred at 0°C for 30 min and at room temperature for a further 2 hours during which time a white precipitate formed. At this time concentrated HCl (20 mL) was added to precipitate excess diisopropylamine. The reaction mixture was filtered and the filtrate was washed with 1.0 M HCl (3 x 50 mL), dried with Na_2SO_4, and concentrated *in vacuo*. The resulting crude solid was washed with ethyl acetate (3 x 20 mL) to yield the pure title compound as a white powder (4.80 g, 83% yield). The ^{1}H and ^{13}C NMR data were in good agreement with those reported in the literature.[1]

2.2. 1-chloro-2,3-bis(dicyclohexylamino)cycloprop-2-en-1-ylium chloride **(2-Cy)**

Dicyclohexylamine (18.6 mL, 60 mmol, 6.0 equiv) was slowly added to a solution of pentachlorocyclpropane (1.3 ml, 10 mmol, 1.0 equiv) in dichloromethane (100 mL) at 0°C. The

resulting solution was stirred at 0°C for 30 min and at room temperature for a further 2 hours during which time a white precipitate formed. At this time concentrated HCl (20 mL) was added to precipitate excess dicyclohexylamine. The reaction mixture was filtered and the filtrate was washed with 1.0 M HCl (3 x 50 mL), dried with Na_2SO_4, and concentrated *in vacuo*. The resulting crude solid was washed with ethyl acetate (3 x 20 mL) to yield the pure title compound as a white powder (6 g, 88% yield). The ^{1}H and ^{13}C NMR data were in good agreement with those reported in the literature.[1]

2.3. 3-imino-bis(diisopropylamino)cycloprop-1-ene hydrochloride (**L1H.HCl**)

Ammonia solution (15 mL) was slowly added to a solution of 2-iPr (3.06 g) in dichloromethane (50 mL) at 0°C. The resulting solution was stirred at 0°C for 30 min. The organic part was separated and the aqueous part was washed with 50 mL DCM. Both organic parts were combined and dried and washed with ethyl acetate to obtain 3-*i*Pr (2 g, 70% yield). The ^{1}H and ^{13}C NMR data were in good agreement with those reported in the literature.[1]

2.4. 3-imino-bis(diisopropylamino)cycloprop-1-ene (**L1H**)

287 mg (1 mmol) of **L1H.HCl** was dissolved in 25 mL of dichloromethane and to it, 5 mL 1M aqueous NaOH solution was added and stirred vigorously at room temperature. The organic part was separated and the aqueous part was washed with 25 mL DCM. Both organic parts were combined and dried and washed with ethyl acetate to obtain **L1H** (200 mg, 80% yield). The ^{1}H and ^{13}C NMR data were in good agreement with those reported in the literature.[1]

Table S1: Crystallographic data for Th1-Th6

	Th1	Th2	Th3	Th4	Th5	Th6
Formulae	$C_{35}H_{59}Cl_2N_3Th$	$C_{47}H_{75}$ Cl_2N_3Th	$C_{42}H_{58}Cl_2N_3Th$	$C_{47}H_{75}Cl_2N_3Th$	$C_{36}H_{61}N_3Th$	$C_{48}H_{77}N_3Th$
Molecular weight	824.79	985.04	824.79	985.04	767.91	928.16
Crystal system	Monoclinic	Monoclinic	Orthorhombic	Triclinic	Monoclinic	Triclinic
Space group	P21/n	C2/c	P212121	P-1	P21/c	P-1
Temperature /K	200	200	200	200	200	200
Wavelength	0.71073	0.71073	0.71073	0.71073	0.71073	0.71073
a/Å	10.3175(8)	17.004(8)	13.3123(11)	10.2480(14)	18.4502(13)	12.6507(11)
b/Å	25.668(2)	19.608(9)	15.8758(12)	11.1407(15)	10.7953(7)	13.1969(11)
c/Å	15.1359(13)	14.420(7)	18.0265(13)	21.501(3)	19.1382(14)	14.8779(14)
α/°	90	90	90	78.610(4)	90	105.465(2)
β/°	108.714(2)	103.056(15)	90	81.793(4)	103.921(2)	103.232(2)
γ/°	90	90	90	77.170(4)	90	98.056(2)
V/ $Å^3$	3796.5(5)	4683(4)	3809.8(5)	4030.1(4)	3699.9(4)	2276.2(3)
Z	4	4	4	2	4	2
Density/gcm^{-1}	1.443	1.397	1.438	1.402	1.379	1.354
Absorption Coefficient	4.094	3.331	4.079	3.342	4.055	3.309
Absorption Correction	Multi-scan	Multi-scan	Multi-scan	Multi-scan	Multi-scan	Multi-scan
F(000)	1656	2008	1656	1004	1552	952
Total no of reflections	6717	3727	6708	7637	6557	8109
Reflections, $I>2\sigma(I)$	5361	2765	5958	3989	4872	6633
Max. 2θ/°	25.140	24.526	25.101	24.527	25.072	25.118
Complete to 2θ(%)	98.9	98.3	98.9	98.4	99.8	99.7
Refinement method	Full-matrix least-squares on F^2	Full-matrix least-squares on F^2	Full-matrix least-squares on F^2	Full-matrix least-squares on F^2	Full-matrix least-squares on F^2	Full-matrix least-squares on F^2
Goof (F^2)	0.989	0.970	0.866	0.819	1.146	1.025
R indices [$I>2\sigma(I)$]	0.0244	0.0673	0.0335	0.0637	0.0451	0.0350
R Indices (all data)	0.0389	0.0900	0.0414	0.1601	0.0707	0.0528
wR2	0.0415	0.1805	0.0588	0.1051	0.0825	0.0686

Table S2: Crystallographic data for Th7-Th12

	**Th7**	**Th8**	**Th9**	**Th10**	**Th11**	**Th12**
Formulae	$C_{35}H_{58}ClN_3Th$	$2(C_{47}H_{74}ClN_3Th)$, $C_4H_{10}O$	$C_{37}H_{65}N_3Th$	$C_{49}H_{81}N_3Th$	$C_{36}H_{62}ClN_3Th$	$C_{48}H_{78}ClN_3Th$
Molecular weight	788.33	1971.27	783.96	944.20	804.37	964.67
Crystal system	Monoclinic	Triclinic	Monoclinic	Triclinic	Monoclinic	triclinic
Space group	C2/c	P1	P21/c	P-1	P21/c	P-1
Temperature/K	200	200	200	200	200	200
Wavelength	0.71073	0.71073	0.71073	0.71073	0.71073	0.71073
a/Å	18.152(2)	12.6980	13.9586(14)	10.1050(6)	13.8811(12)	10.1126(17)
b/Å	14.0068(16)	14.0258(5)	14.0484(13)	13.6150(11)	14.0424(12)	13.574(2)
c/Å	30.448(5)	14.8586(6)	19.6416(18)	18.6434(12)	19.6757(16)	18.620(3)
α/°	90	69.007(4)	90	72.216(3)	90	90
β/°	106.070(3)	74.326(4)	93.945(3)	83.645(2)	93.923(2)	83.724(4)
γ/°	90	80.972(3)	90	71.2770(10)	90	71.304(4)
V/ $Å^3$	7439.0(17)	2373.45(17)	3842.5(6)	2312.9(3)	3826.3(6)	2303.8(7)
Z	8	1	4	2	4	2
Density/gcm^{-1}	1.408	-	1.355	1.356	1.396	1.3905
Absorption Coefficient	4.105	3.233	3.906	3.258	3.992	3.328
Absorption Correction	Multi-scan	Analytical	Multi-scan	Multi-scan	Multi-scan	Multi-scan
F(000)	3168	1010	1592	972	1624	975
Total no of reflections	6554	16826	6824	8088	6773	8386
Reflections, $I>2\sigma(I)$	4228	13899	4688	6668	4558	6379
Max. 2θ/°	25.157	29.998	25.072	25.109	25.095	25.87
Complete to 2θ(%)	98.2	99.7	99.9	98.1	99.5	93.7
Refinement method	Full-matrix least-squares on F^2	Full-matrix least-squares on F^2	Full-matrix least-squares on F^2	ll-matrix least-squares on F^2	Full-matrix least-squares on F^2	Full-matrix least-squares on F^2
Goof (F^2)	0.976	1.016	0.990	0.962	1.001	0.897
R indices [$I>2\sigma(I)$]	0.0432	0.0511	0.0356	0.0323	0.0470	0.0559
R Indices (all data)	0.0884	0.0674	0.0722	0.0455	0.0742	0.1100
wR2	0.0740	0.1289	0.0695	0.0621	0.1089	0.1137

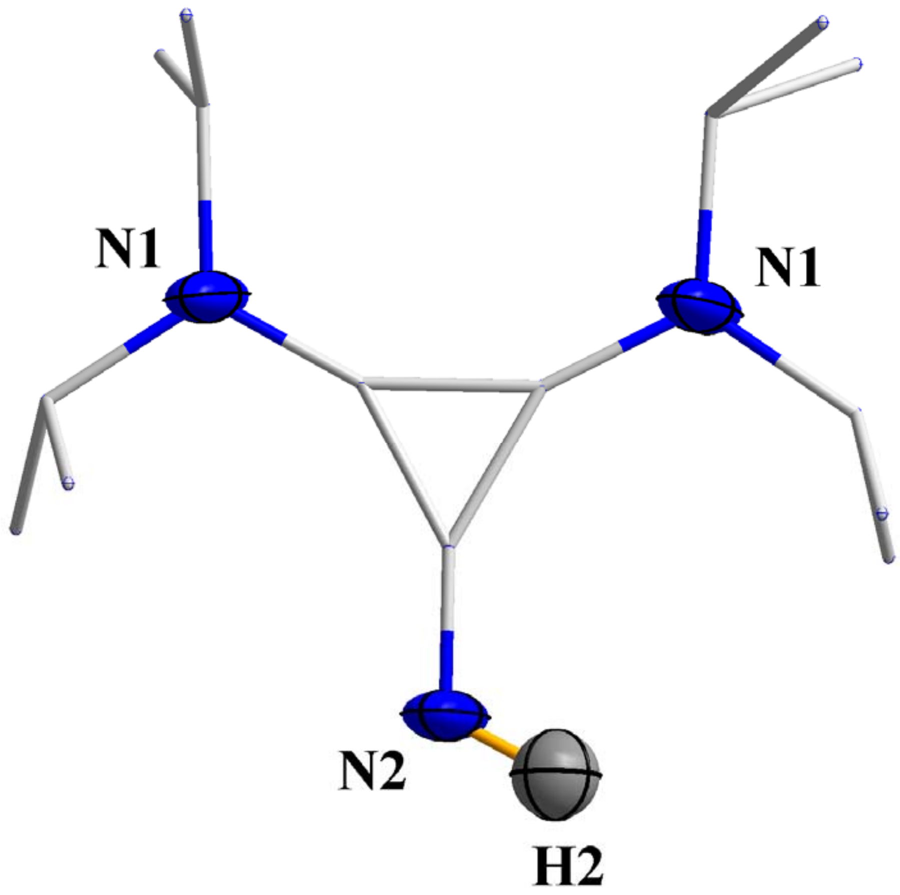

a)

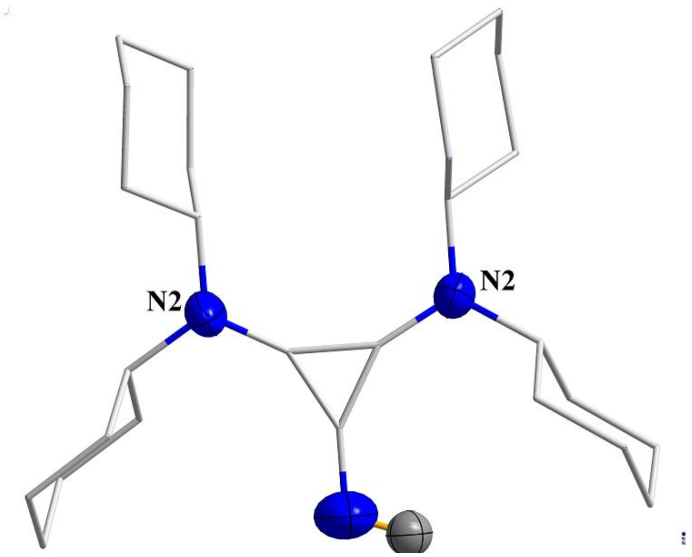

b)

Figure S1: ORTEP diagram of a) **L1H** and b) **L2H**. Hydrogen atom except imine hydrogen is omitted for clarity.

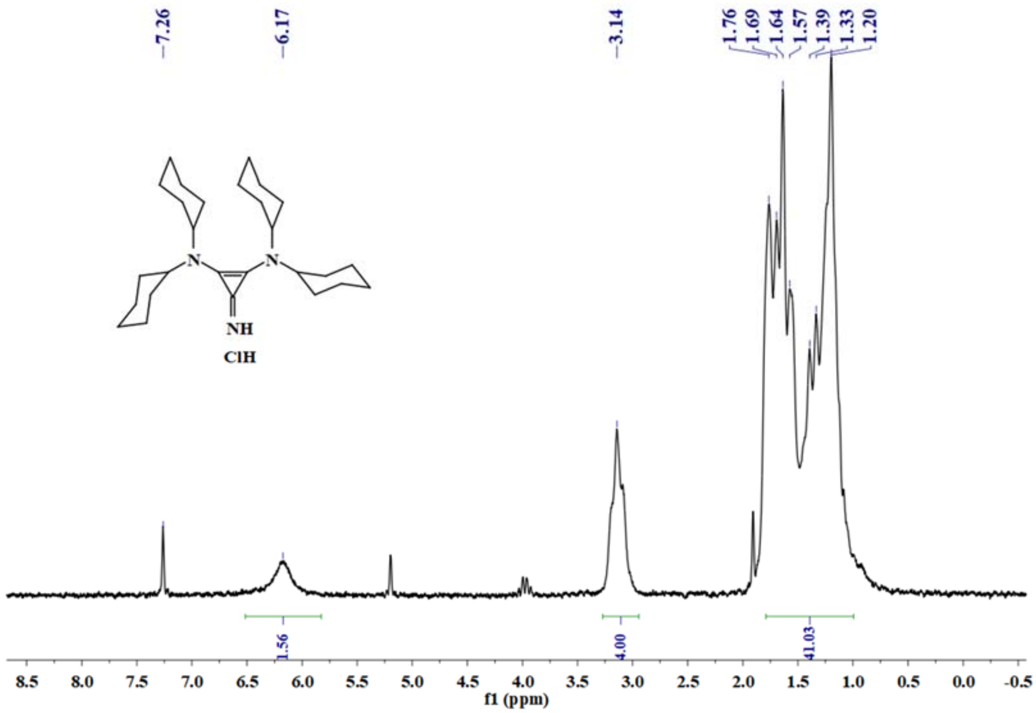

Figure S2. ^{1}H NMR spectrum of **L2H.HCl** in $CDCl_3$.

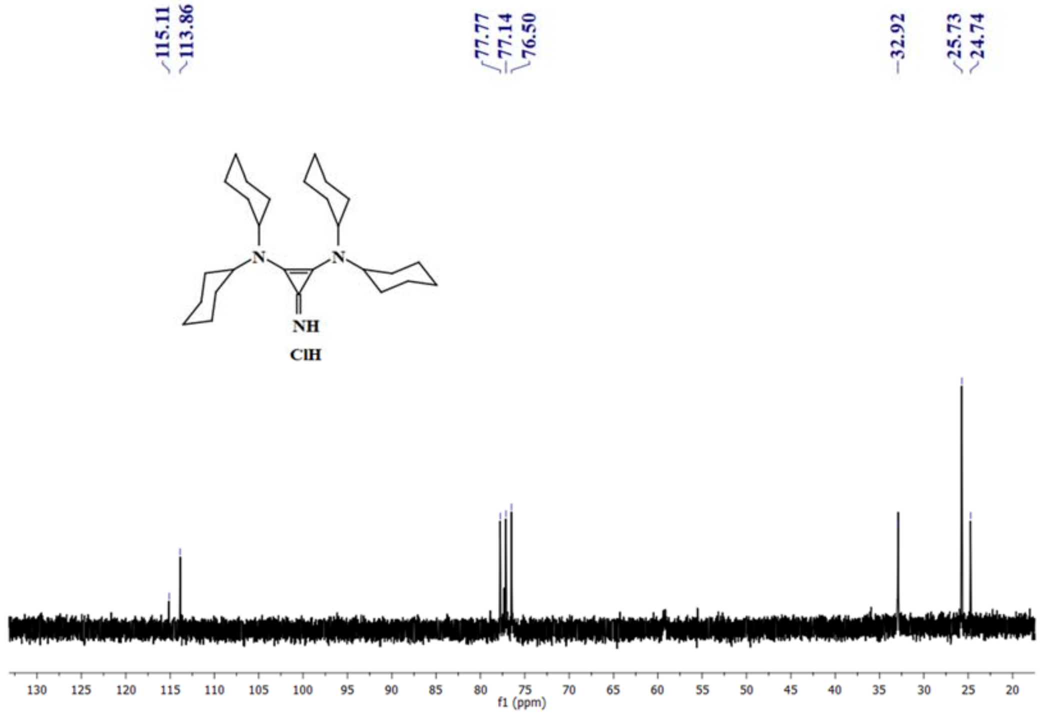

Figure S3. ^{13}C NMR spectrum of **L2H.HCl** in $CDCl_3$.

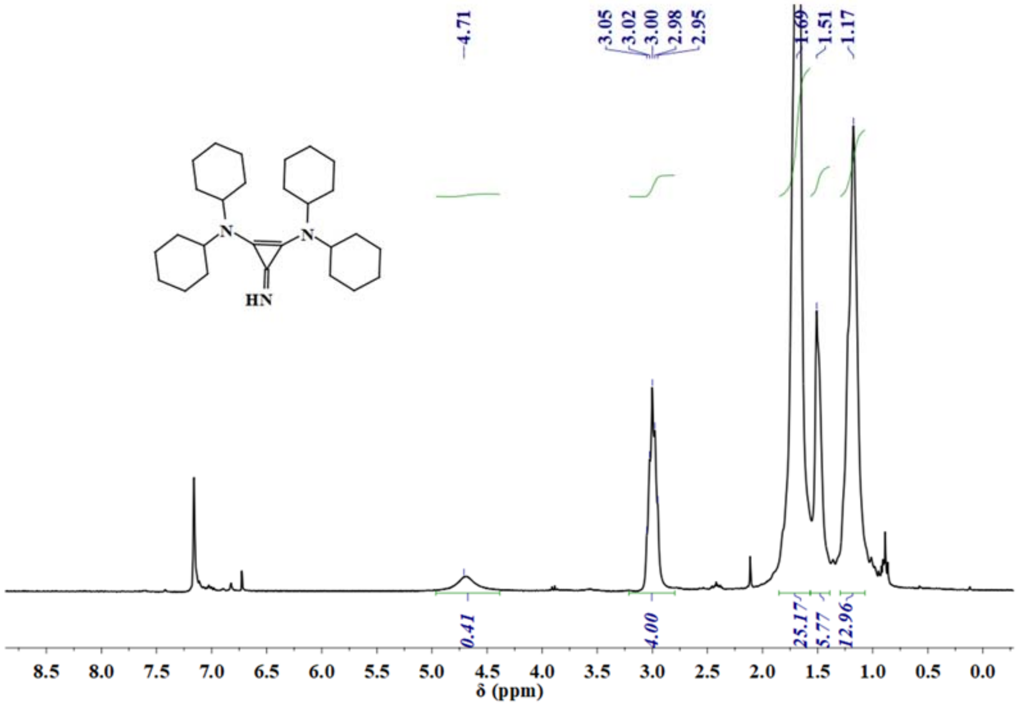

Figure S4. ^{1}H NMR spectrum of **L2H** in C_6D_6.

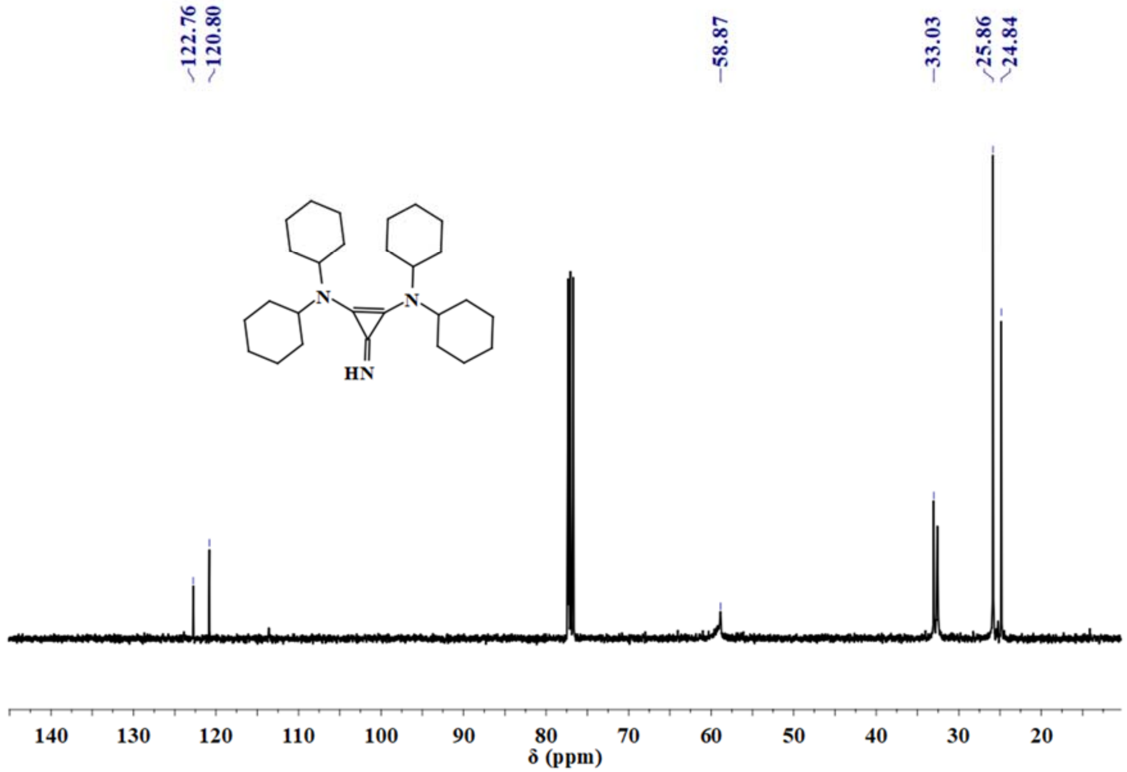

Figure S5. ^{13}C NMR spectrum of **L2H** in $CDCl_3$.

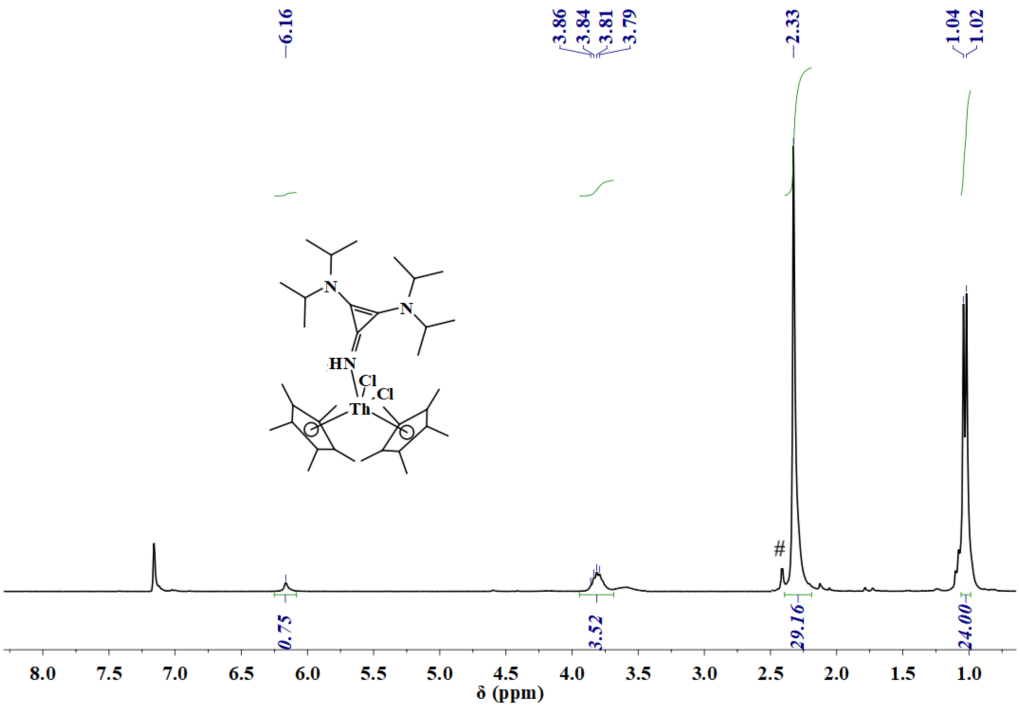

Figure S6. ^{1}H NMR spectrum of **Th1** in C_6D_6. (# indicates impurities of unreacted metal precursor)

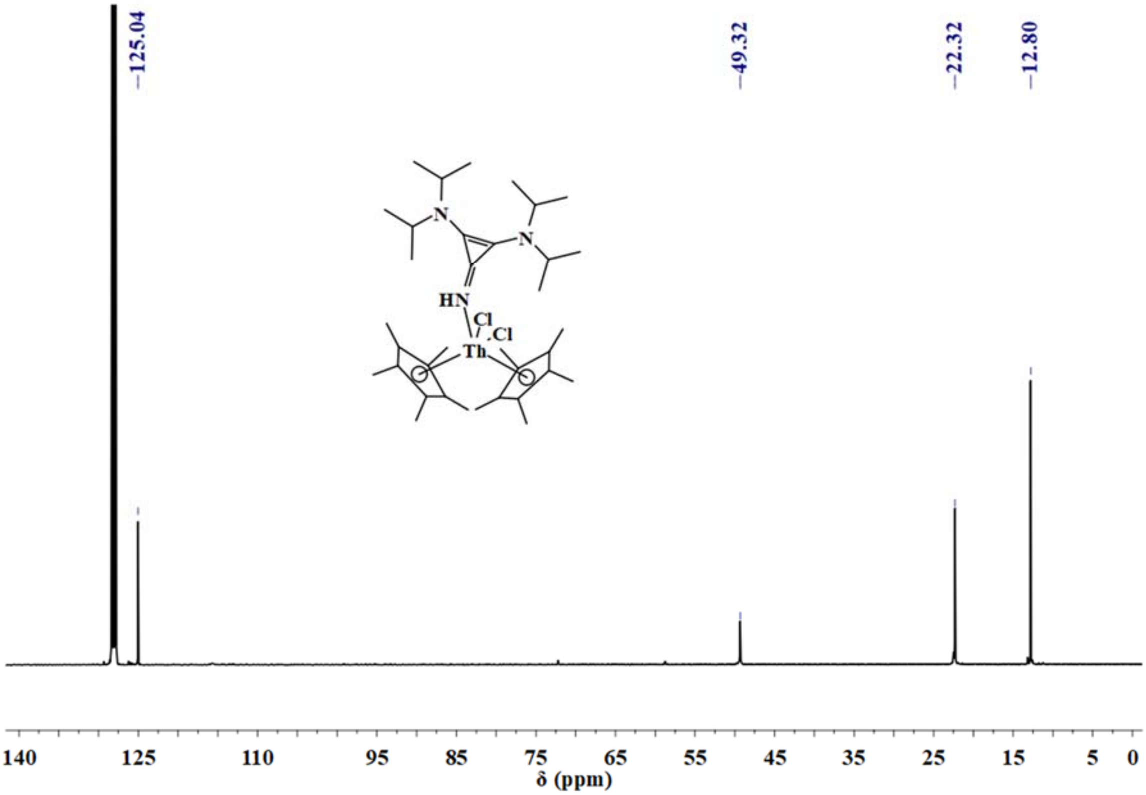

Figure S7. ^{13}C NMR spectrum of **Th1** in C_6D_6.

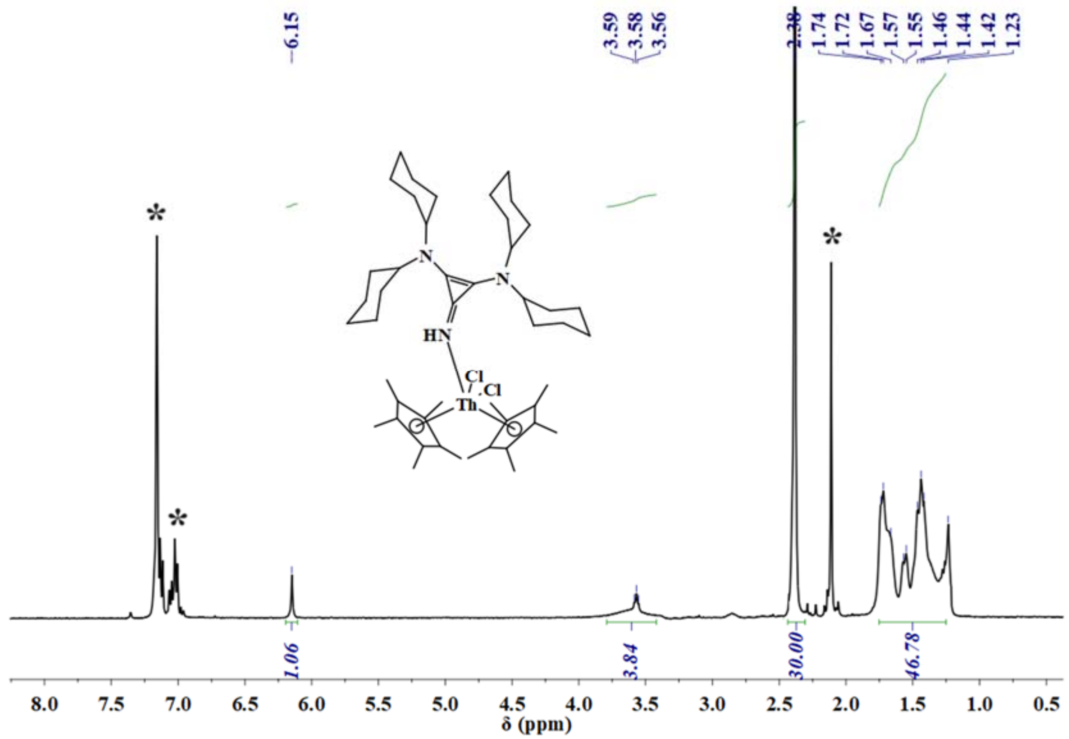

Figure S8. ^{1}H NMR spectrum of **Th2** in C_6D_6. * Indicates toluene.

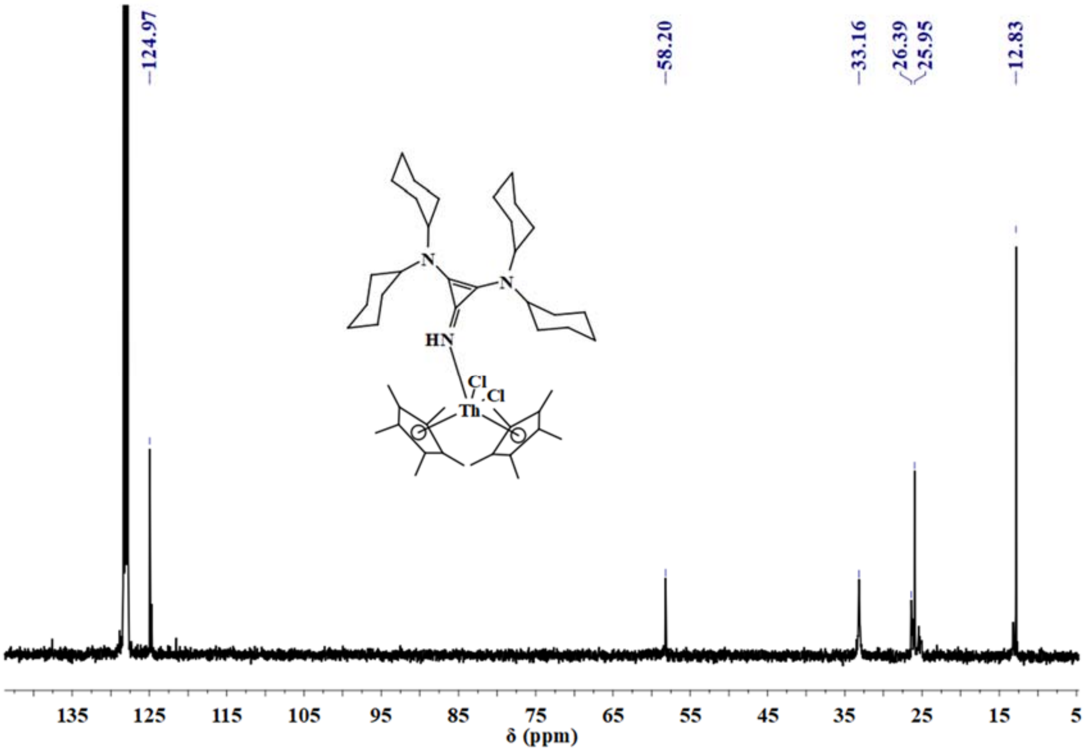

Figure S9. ^{13}C NMR spectrum of **Th2** in C_6D_6.

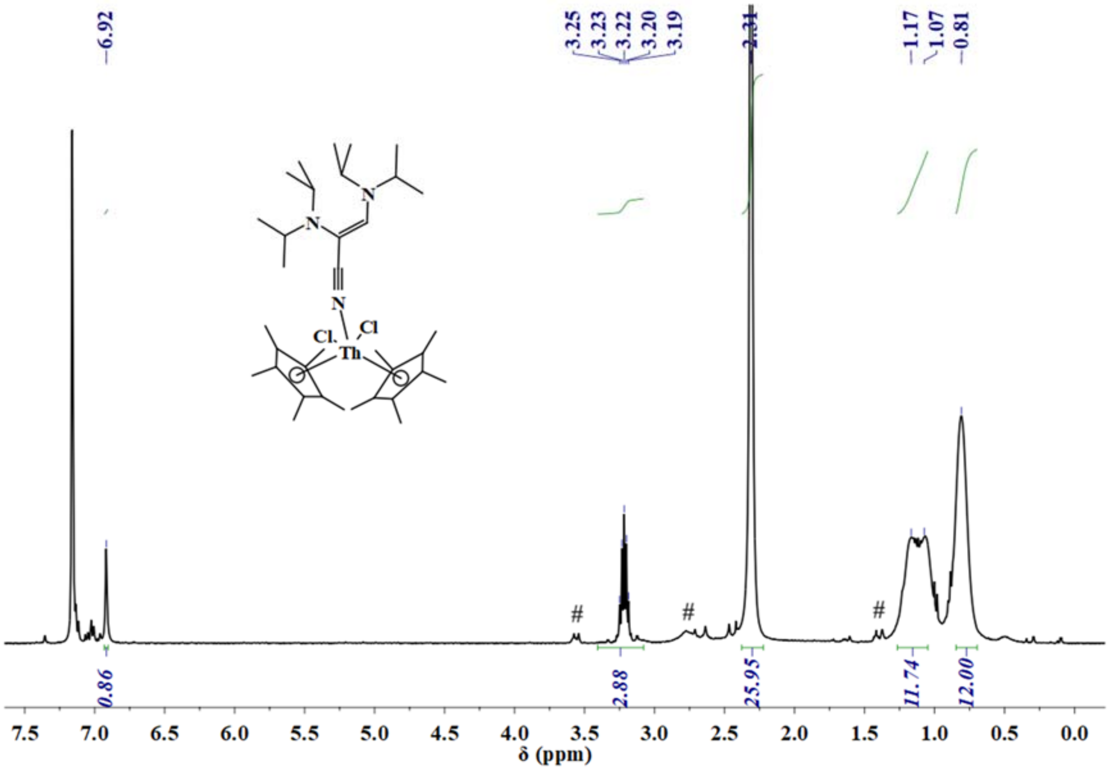

Figure S10. ^{1}H NMR spectrum of **Th3** in C_6D_6. (# indicates impurities)

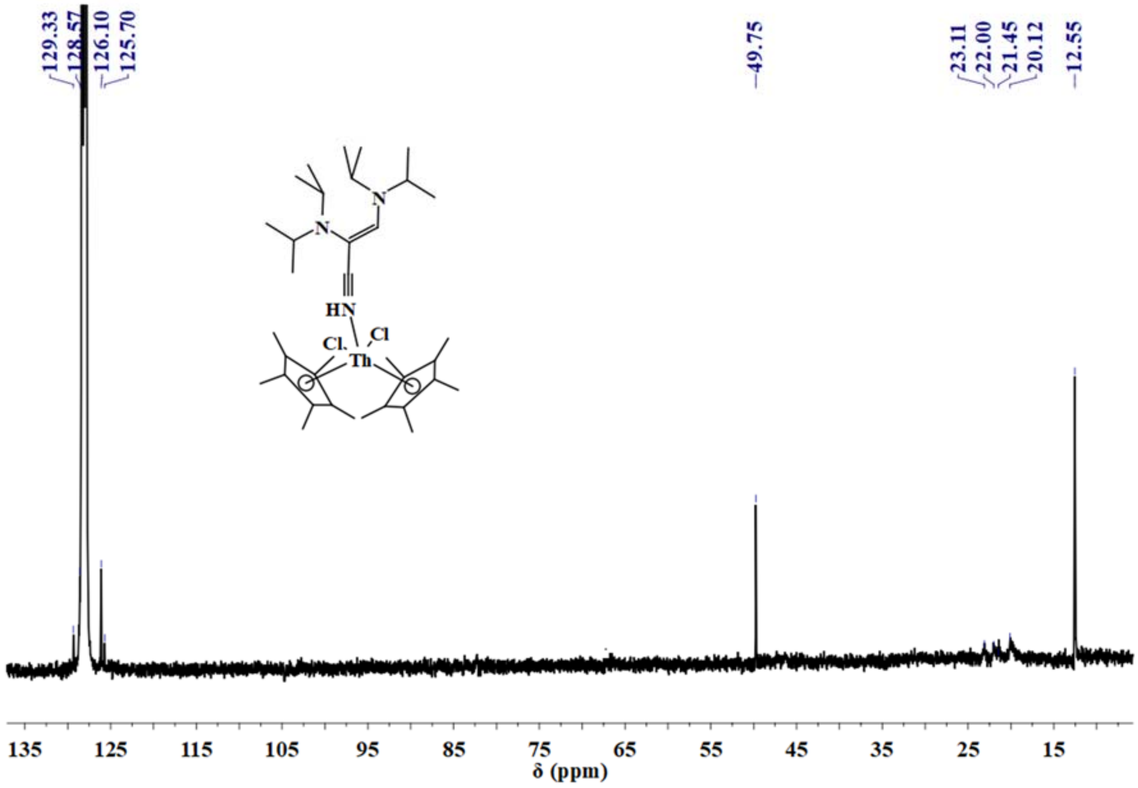

Figure S11. ^{13}C NMR spectrum of **Th3** in C_6D_6.

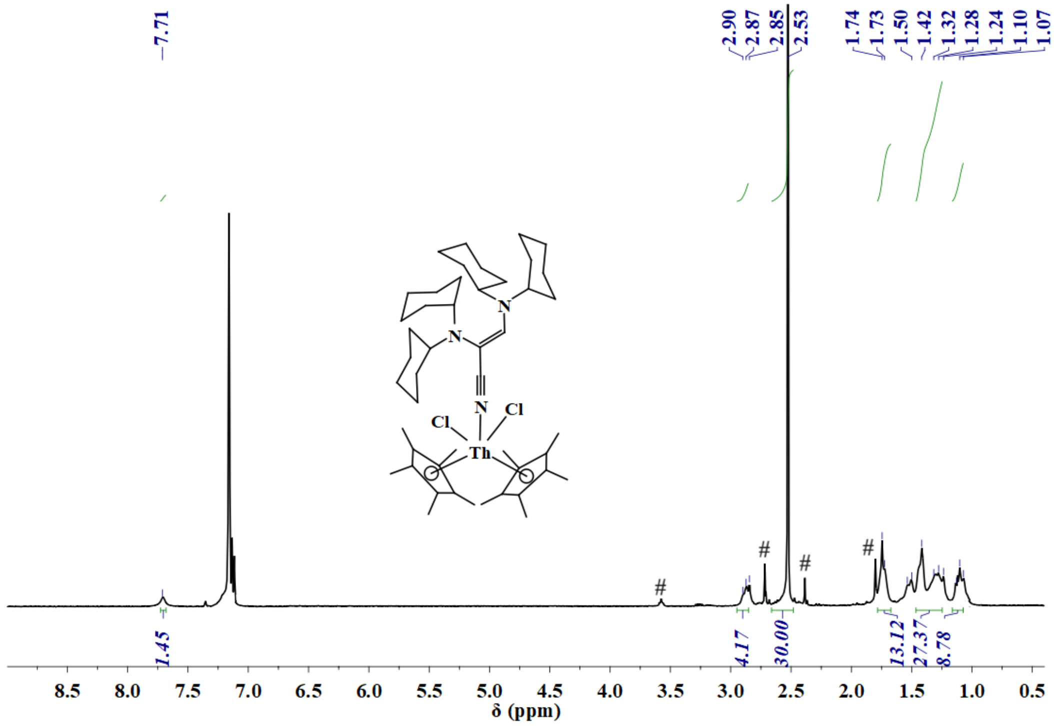

Figure S12. ^{1}H NMR spectrum of **Th4** in C_6D_6. (# indicates impurities)

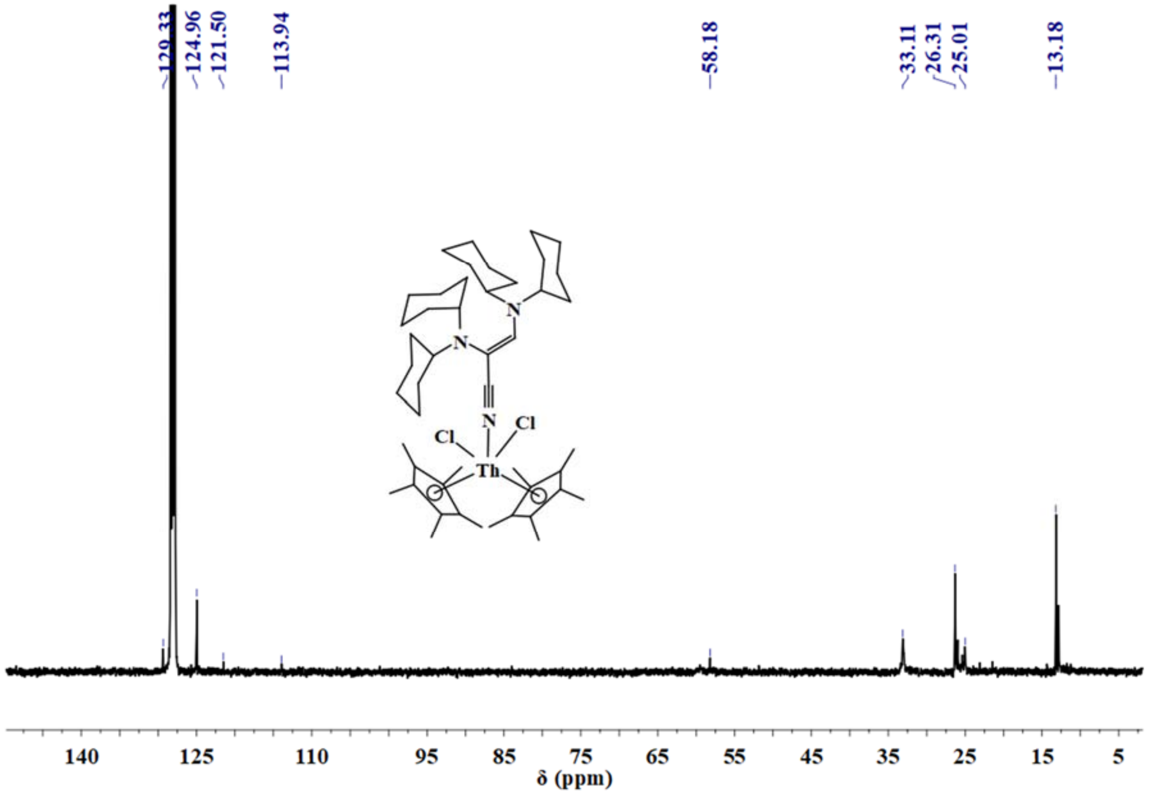

Figure S13. ^{13}C NMR spectrum of **Th4** in C_6D_6.

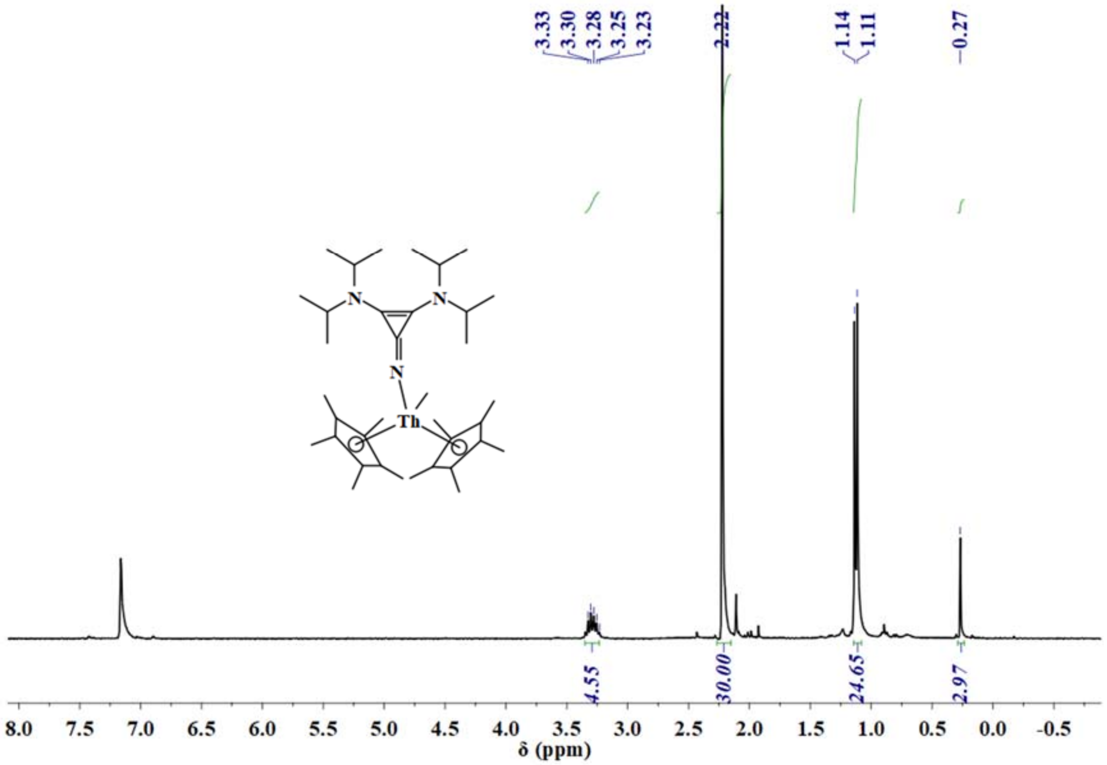

Figure S14. ^{1}H NMR spectrum of **Th5** in C_6D_6.

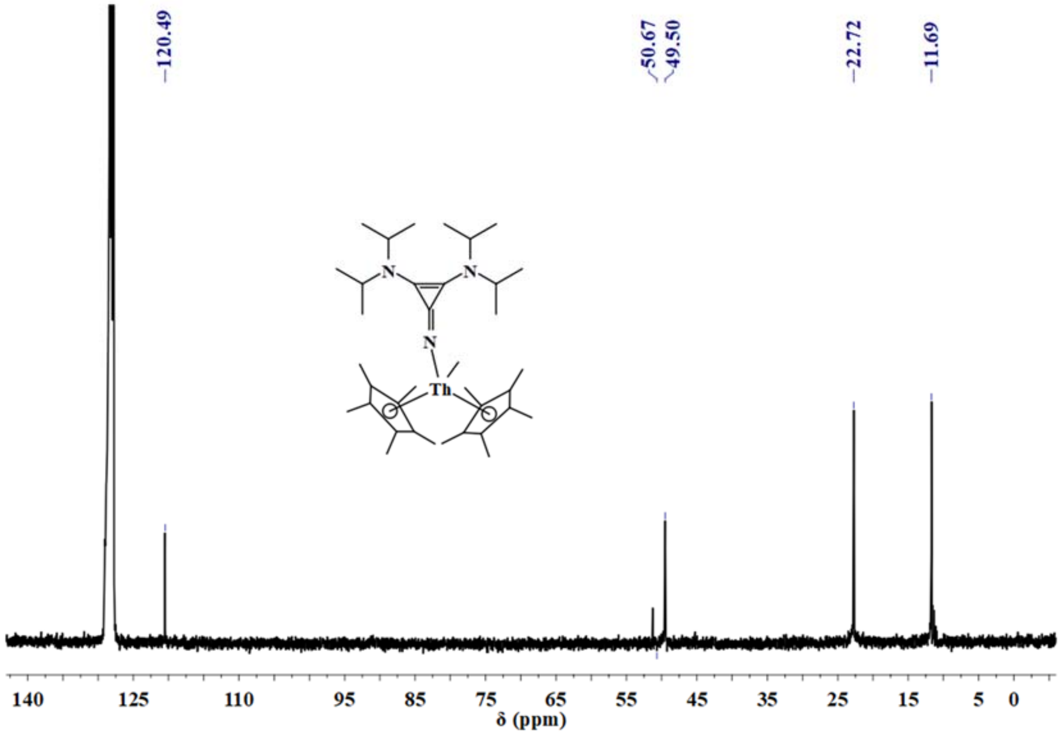

Figure S15. ^{13}C NMR spectrum of **Th5** in C_6D_6.

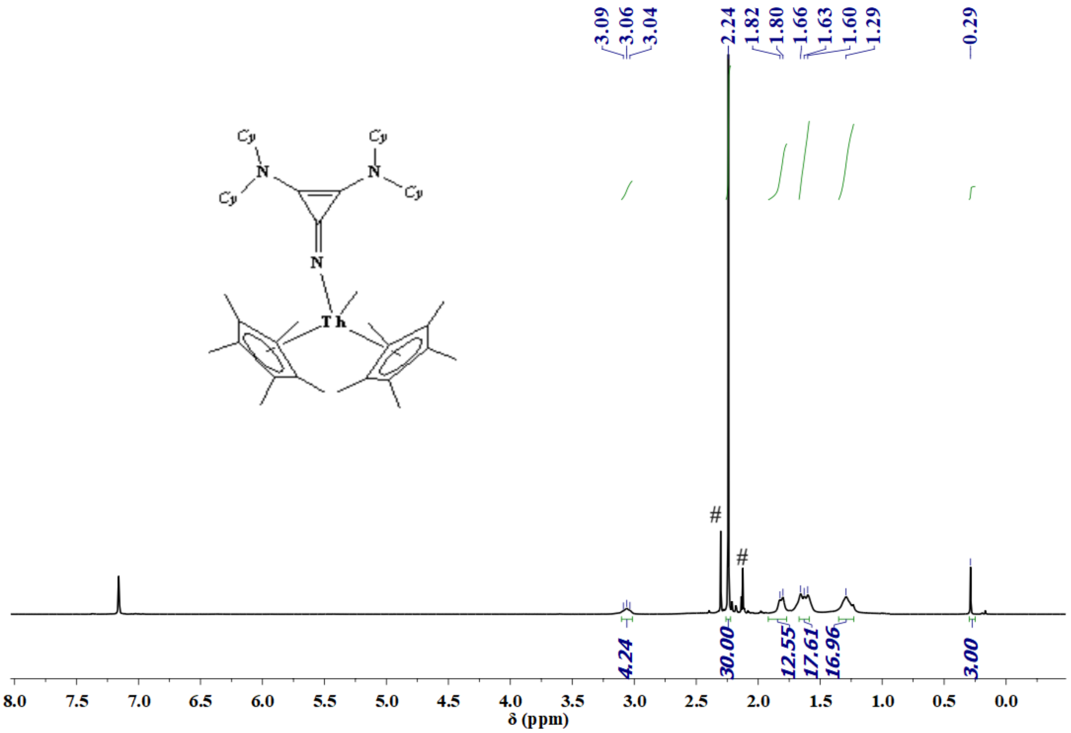

Figure S16. ^{1}H NMR spectrum of **Th6** in C_6D_6. (# indicates impurities)

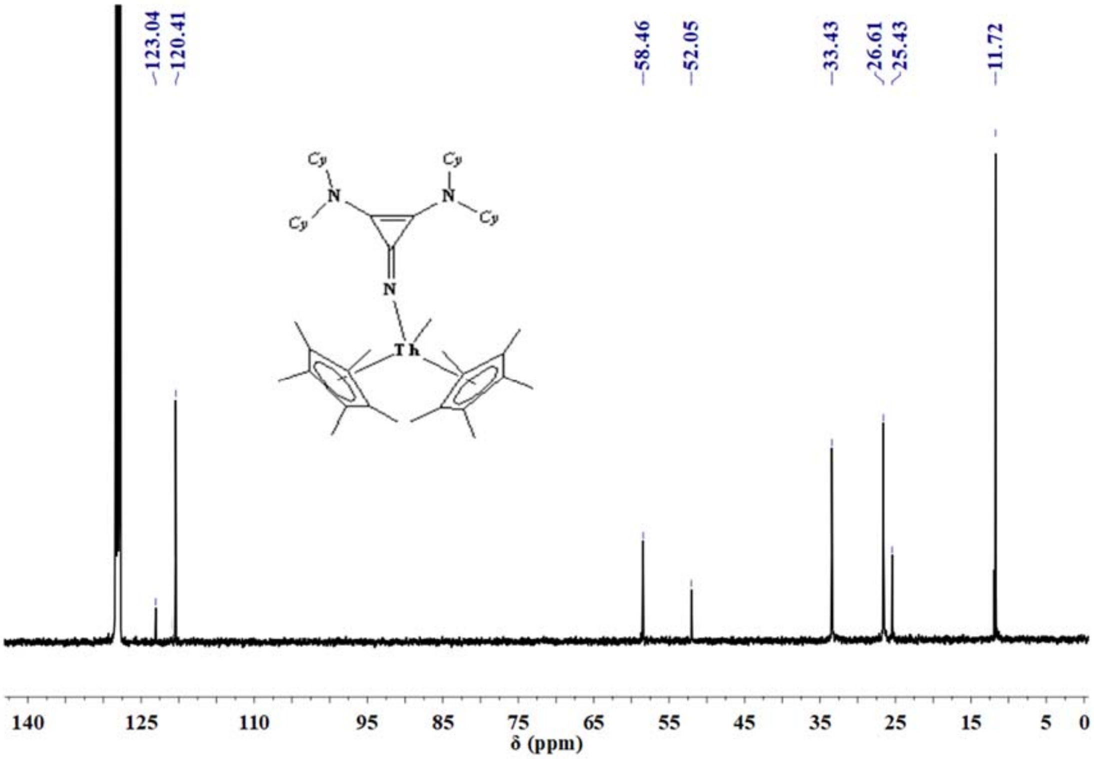

Figure S17. ^{13}C NMR spectrum of **Th6** in C_6D_6.

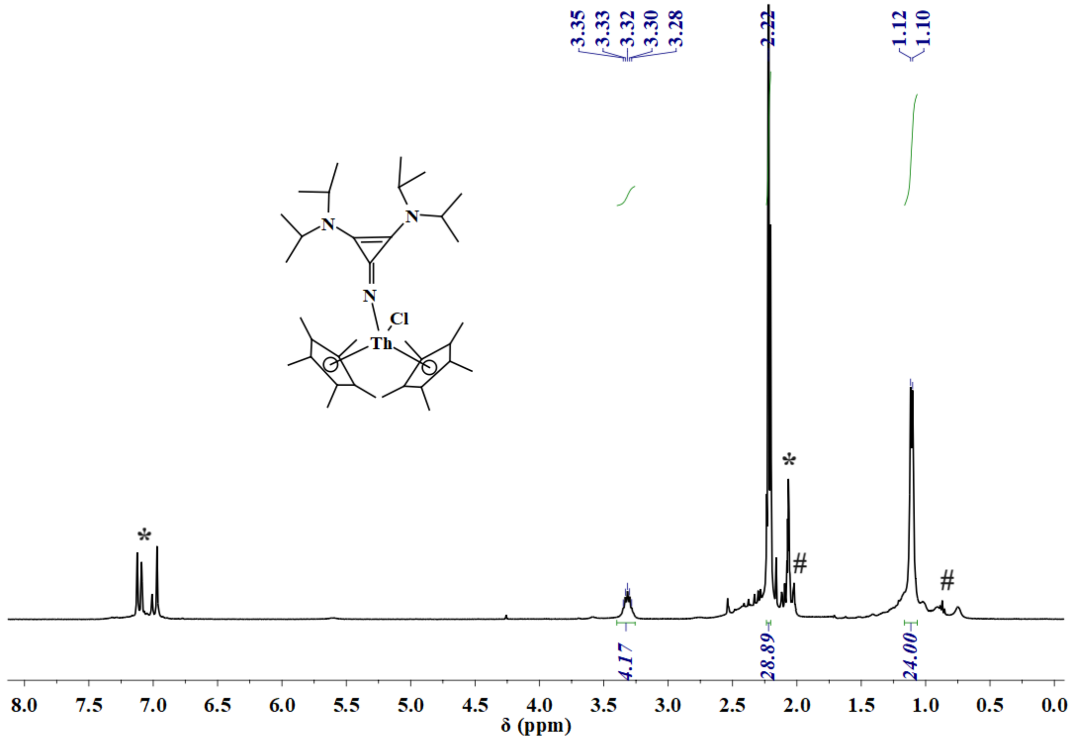

Figure S18. ^{1}H NMR spectrum of **Th7** in toluene-d8. (* indicates residual solvent peak, # indicates impurities.)

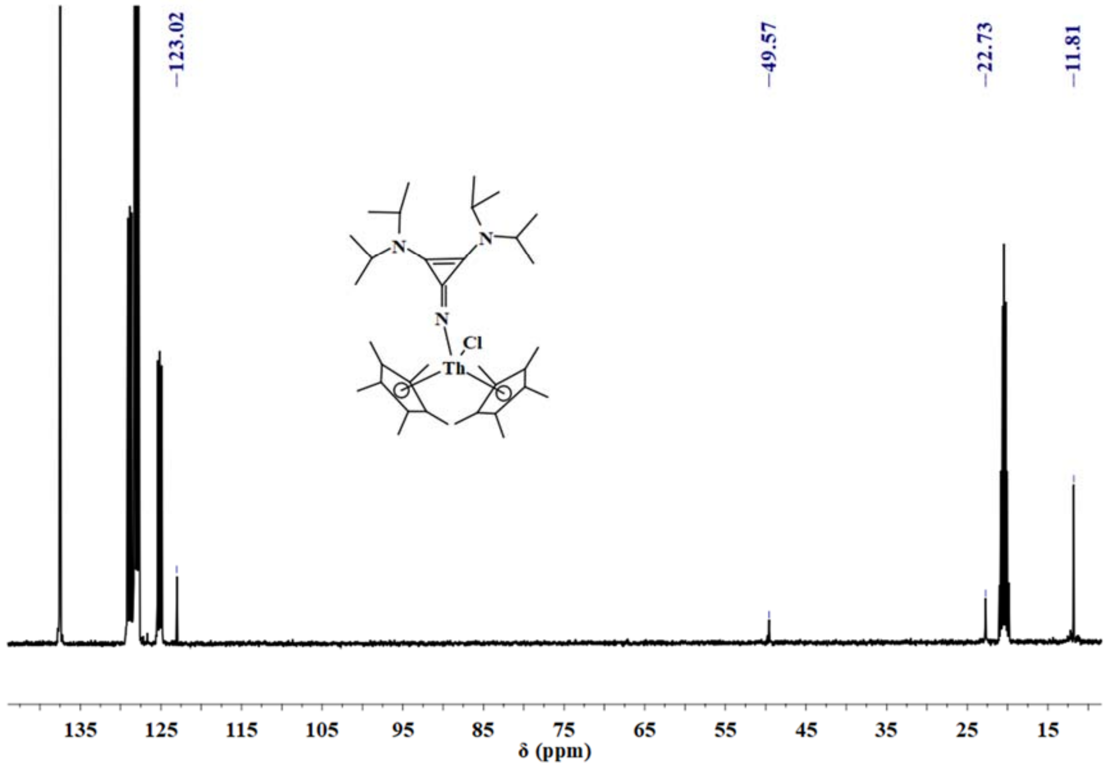

Figure S19. ^{13}C NMR spectrum of **Th7** in toluene-d8.

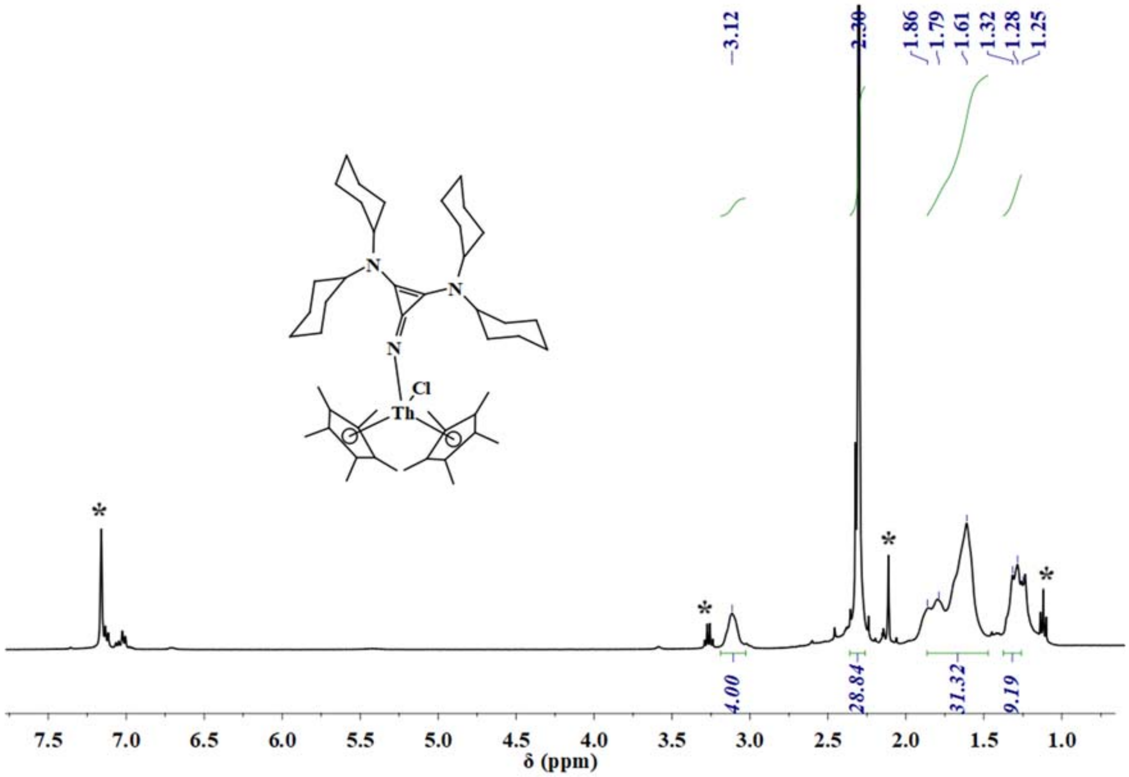

Figure S20. ^{1}H NMR spectrum of **Th8** in C_6D_6. (* indicates residual ether and toluene solvent.)

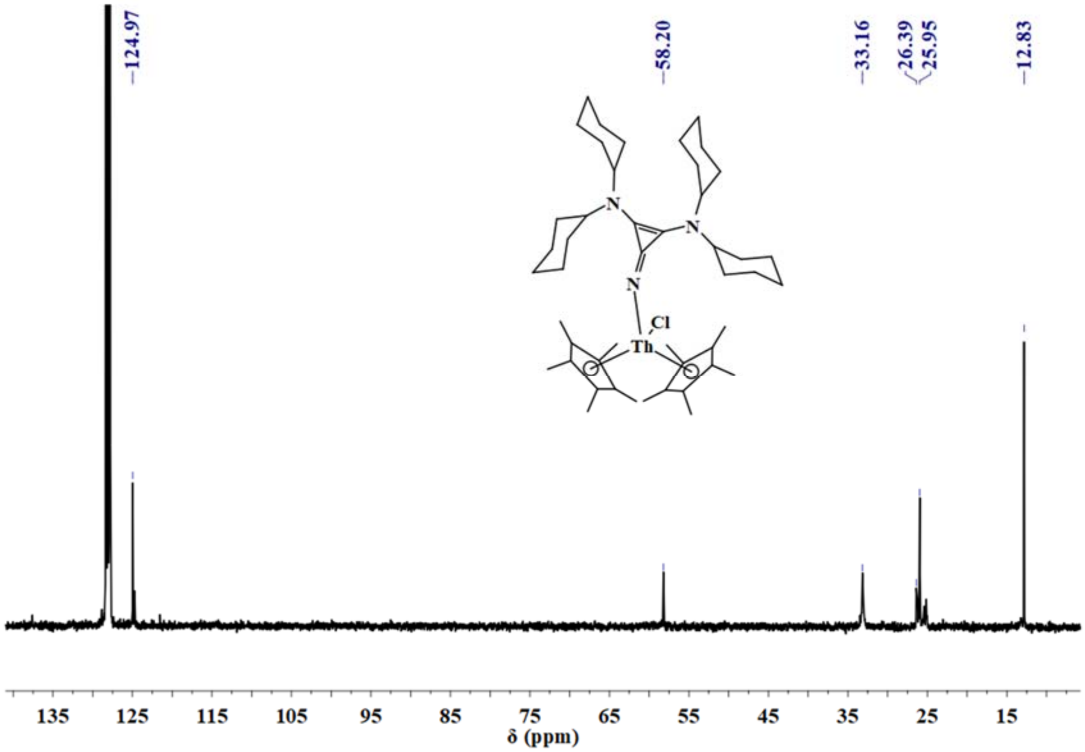

Figure S21. ^{1}H NMR spectrum of **Th8** in C_6D_6.

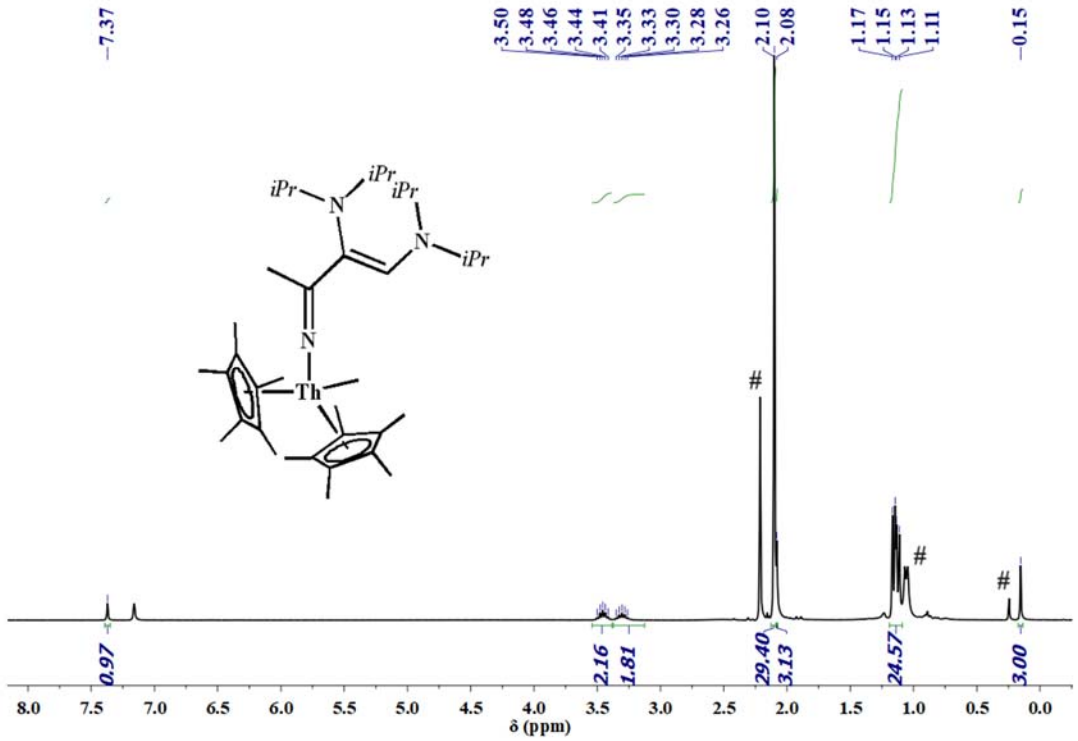

Figure S22. ^{1}H NMR spectrum of **Th9** in C_6D_6. # indicates **Th5**.

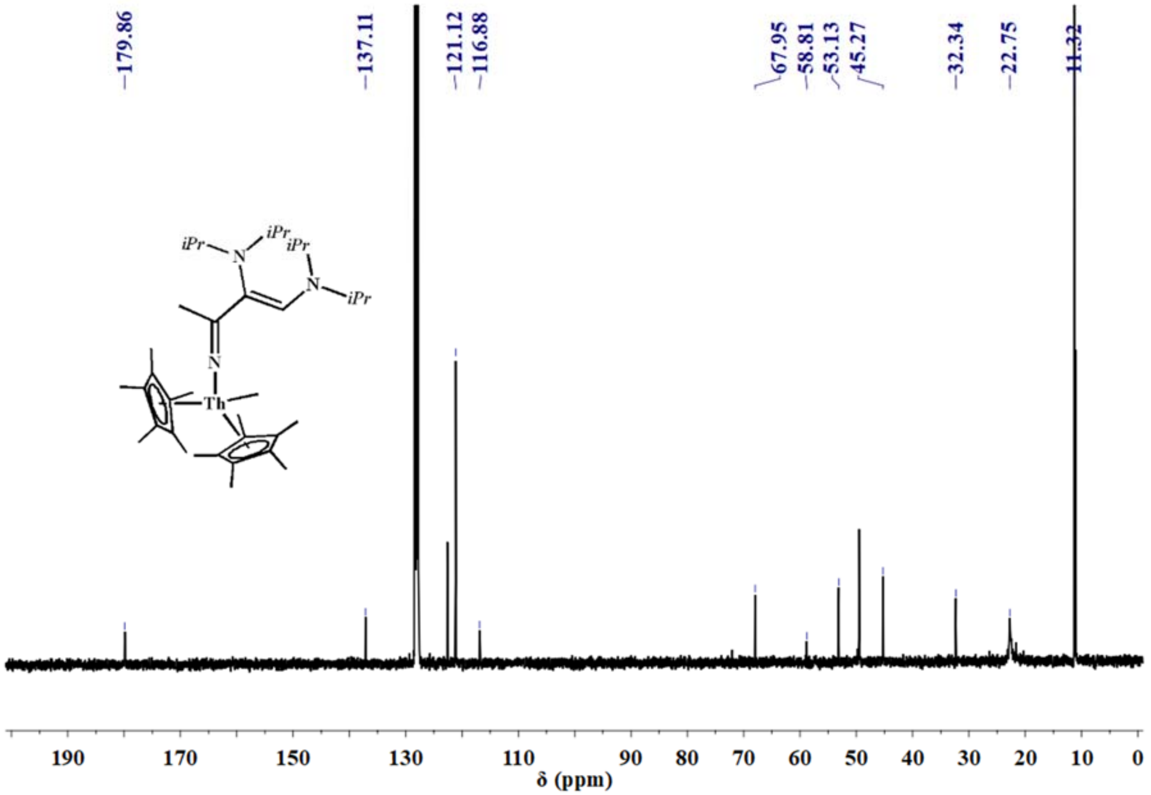

Figure S23. ^{13}C NMR spectrum of **Th9** in C_6D_6.

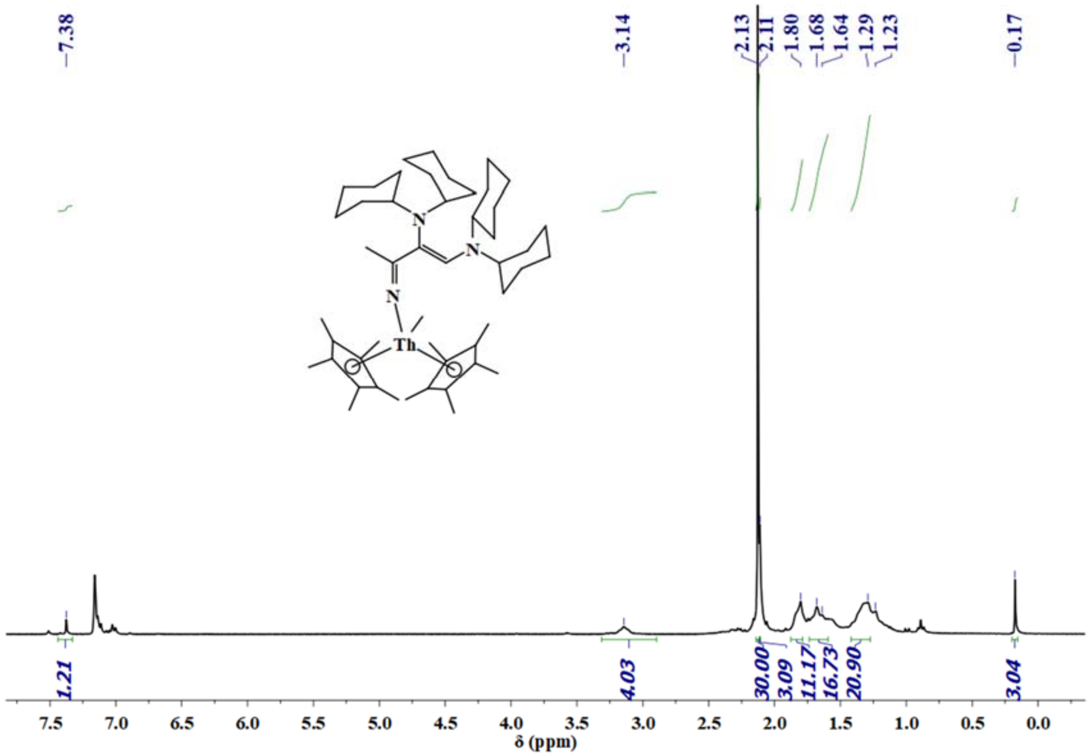

Figure S24. ^{1}H NMR spectrum of **Th10** in C_6D_6.

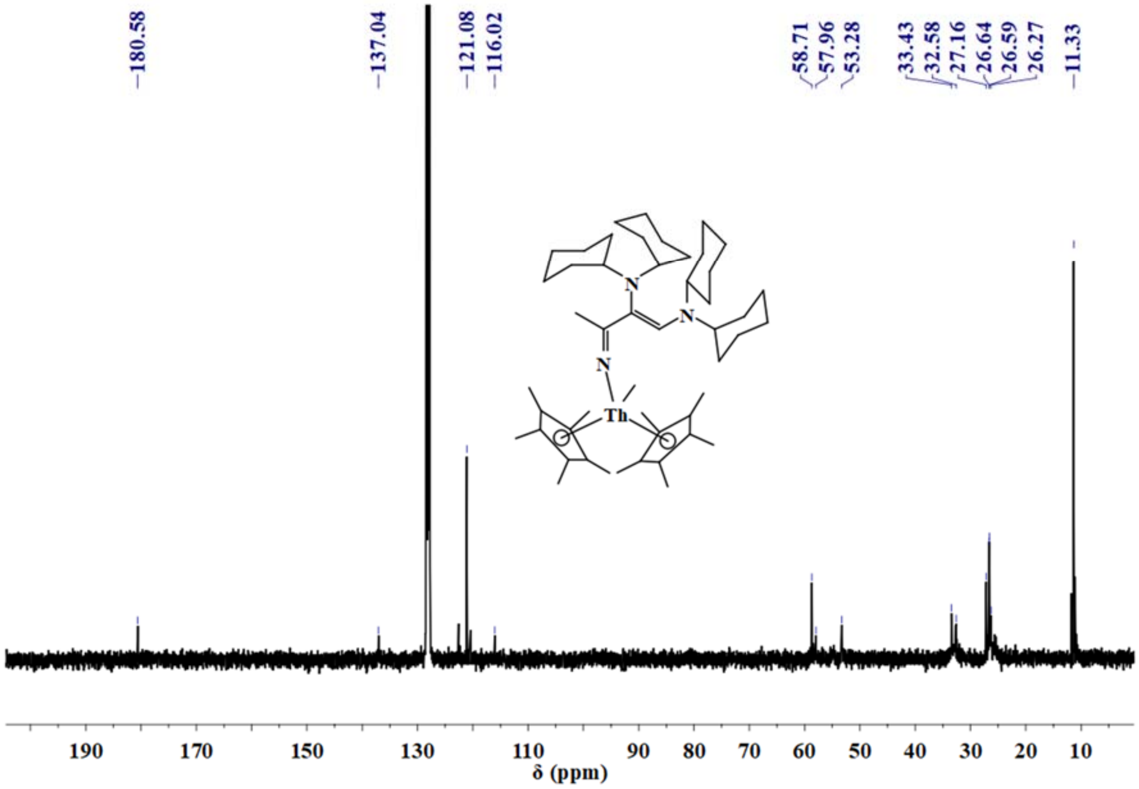

Figure S25. ^{1}H NMR spectrum of **Th10** in C_6D_6.

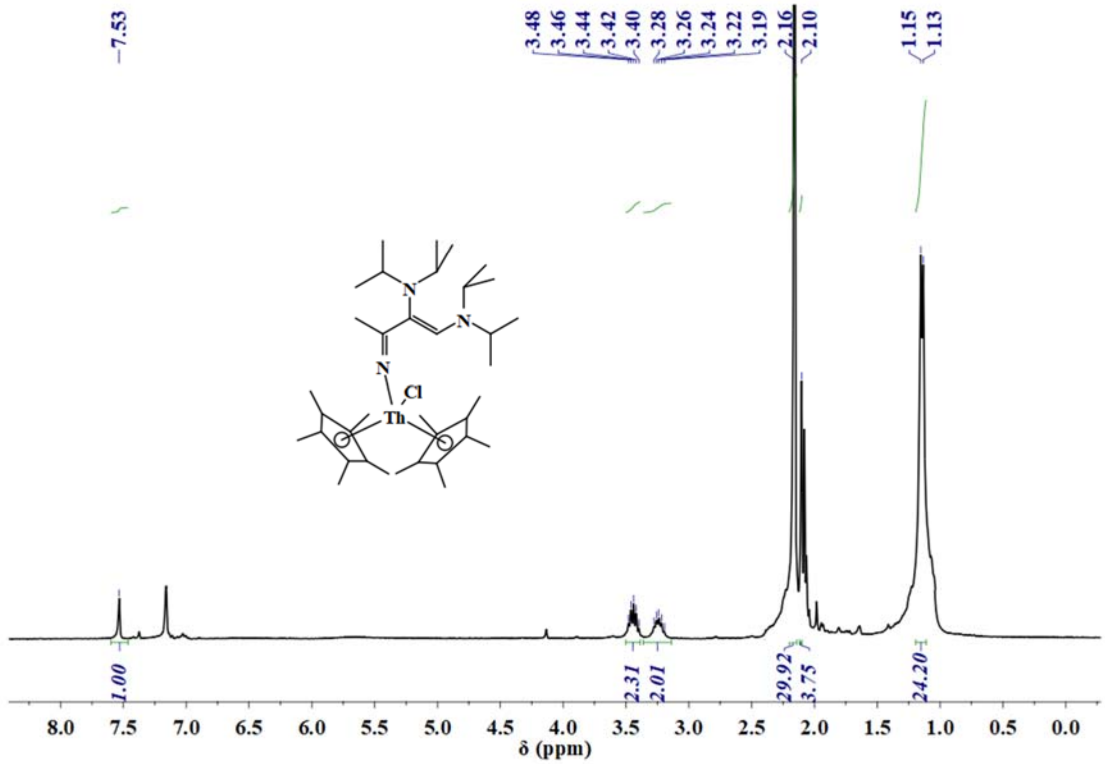

Figure S26. ^{1}H NMR spectrum of **Th11** in C_6D_6.

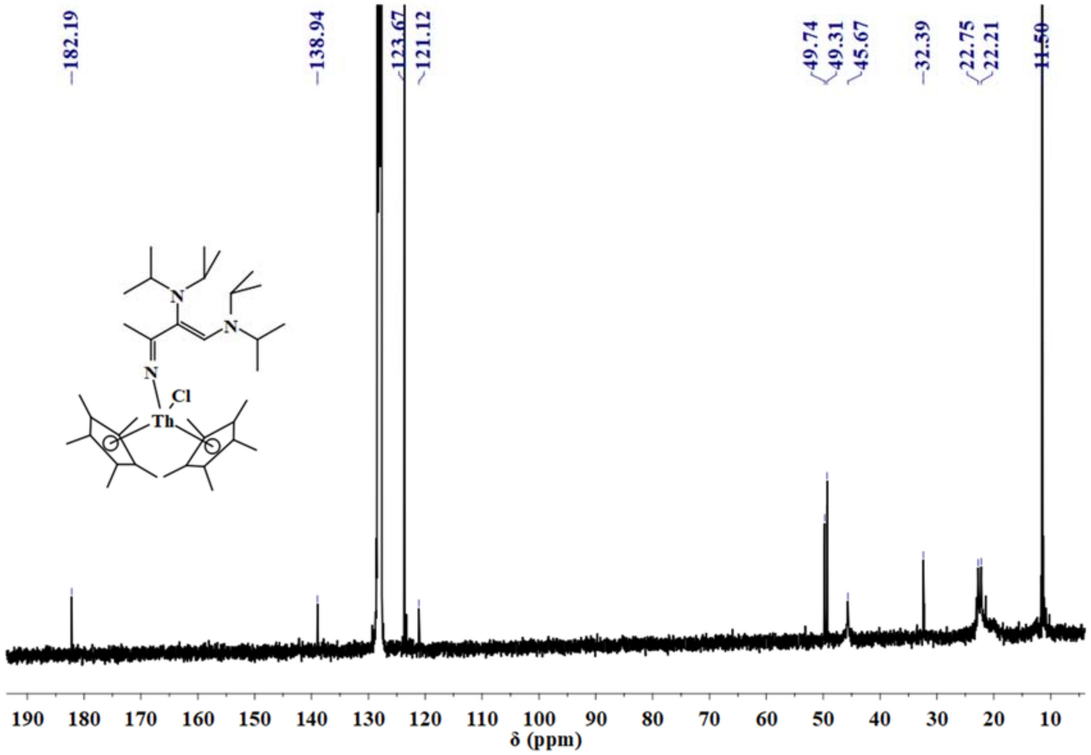

Figure S27. ^{13}C NMR spectrum of **Th11** in C_6D_6.

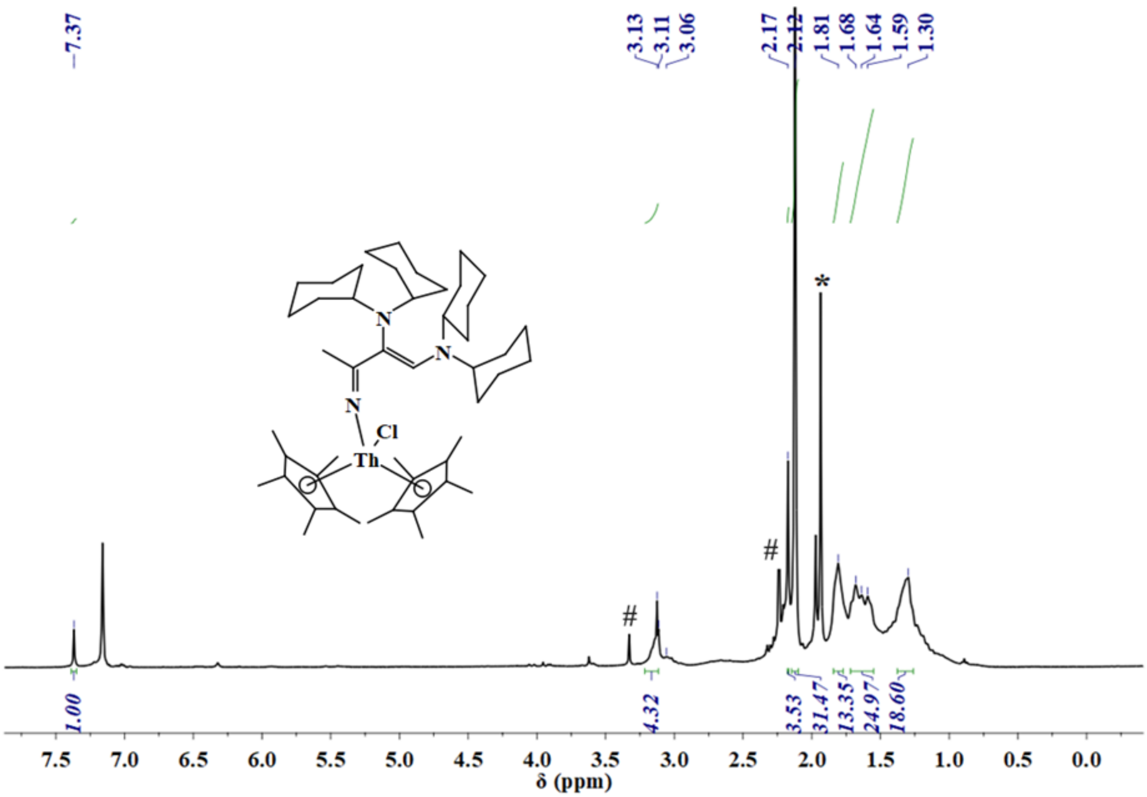

Figure S28. ^{1}H NMR spectrum of **Th12** in C_6D_6. (* indicates residual unreacted metal precursor, # indicates impurities.)

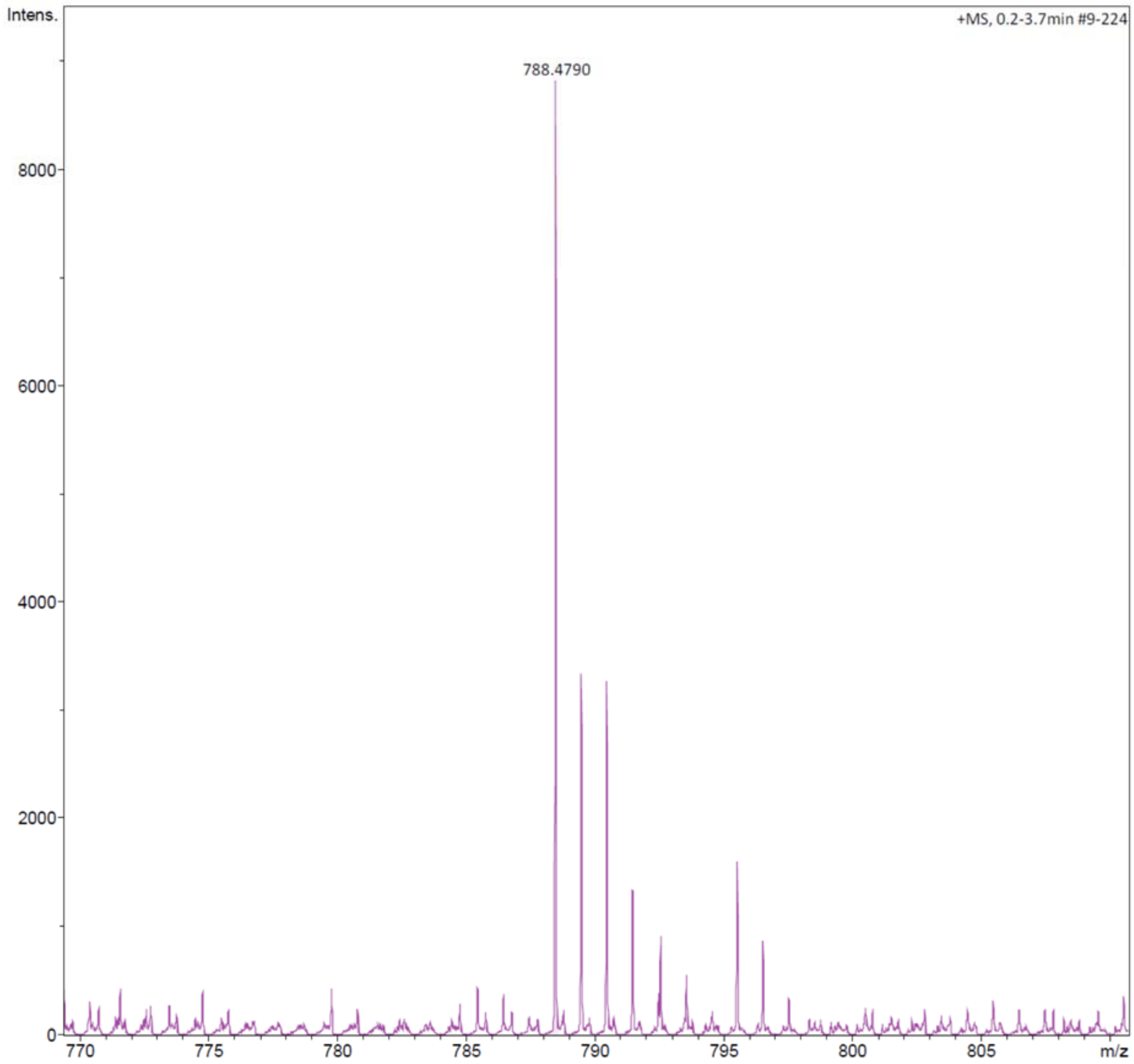

Figure S29. APCI mass spectrum of **Th1**.

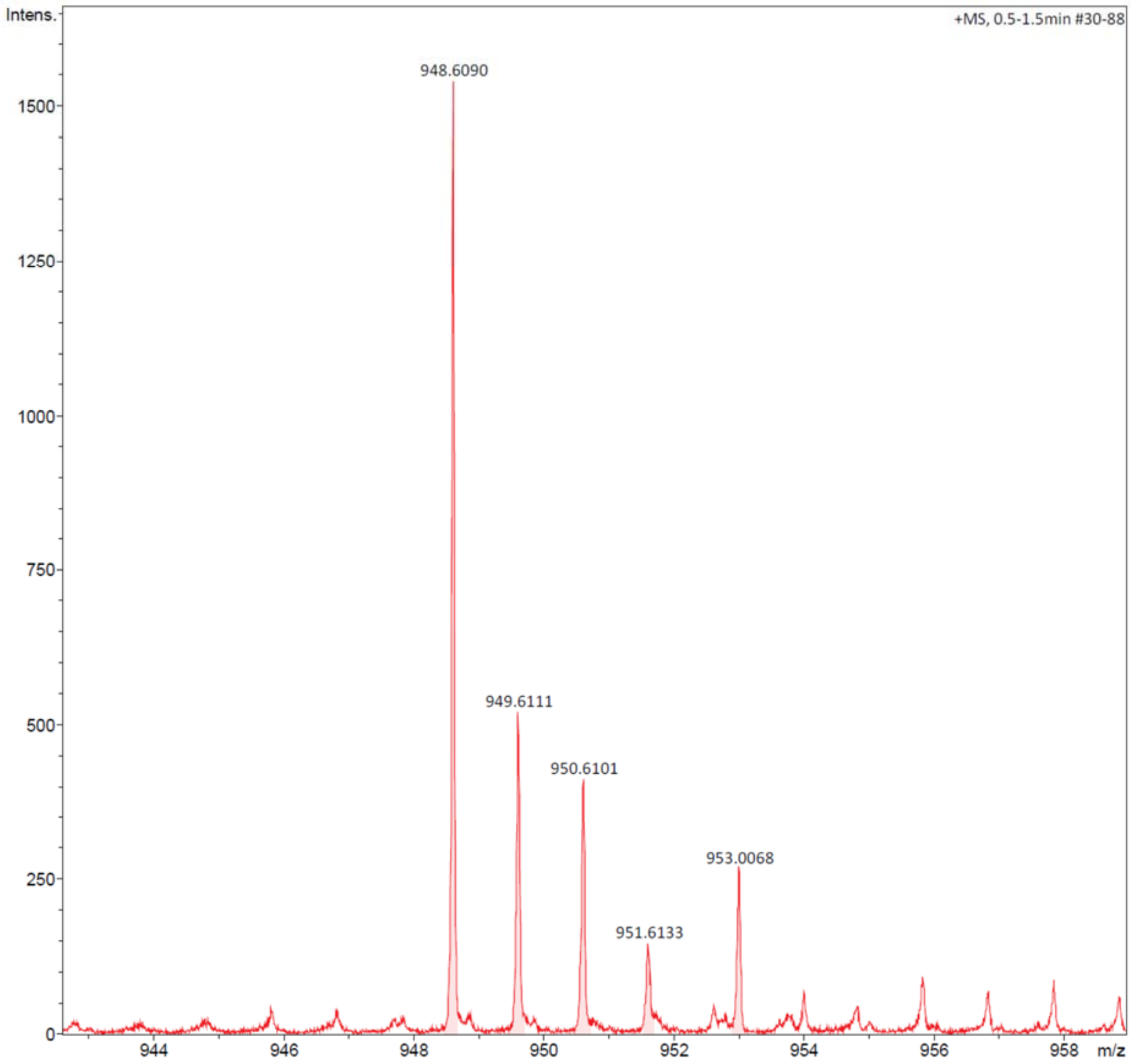

Figure S30. APCI mass spectrum of **Th2**.

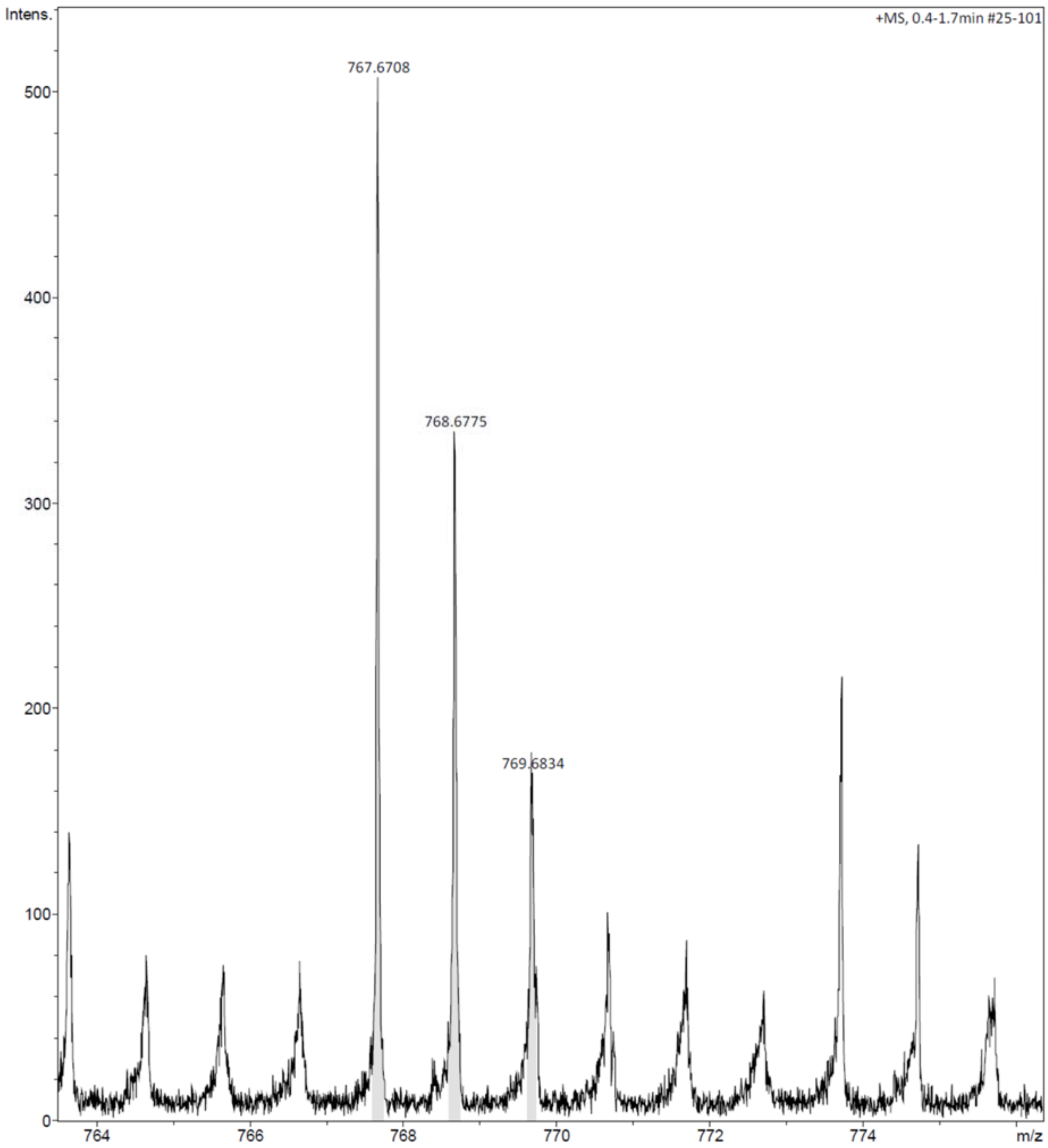

Figure S31. APCI mass spectrum of **Th5**.

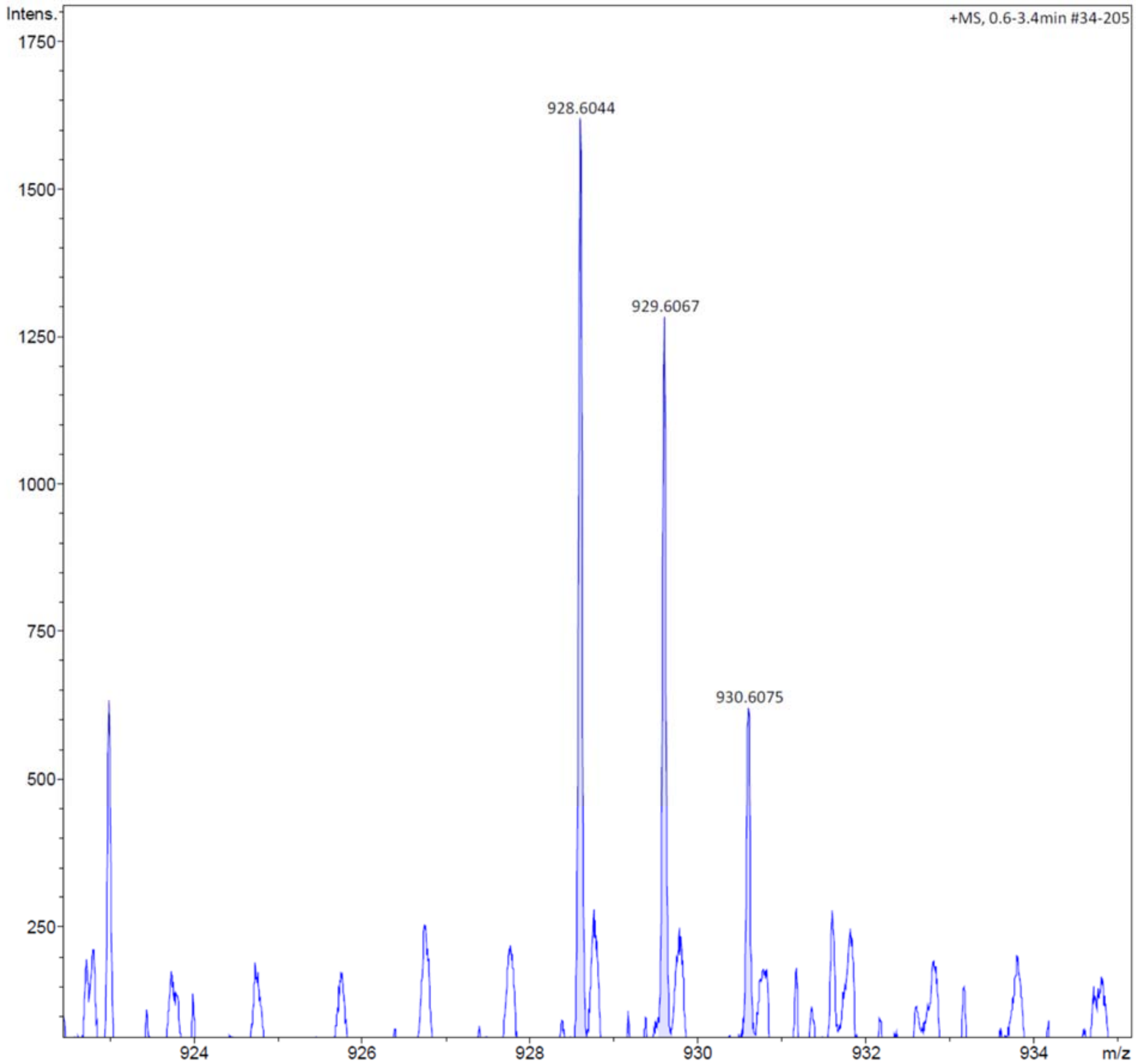

Figure S32. APCI mass spectrum of **Th6**.

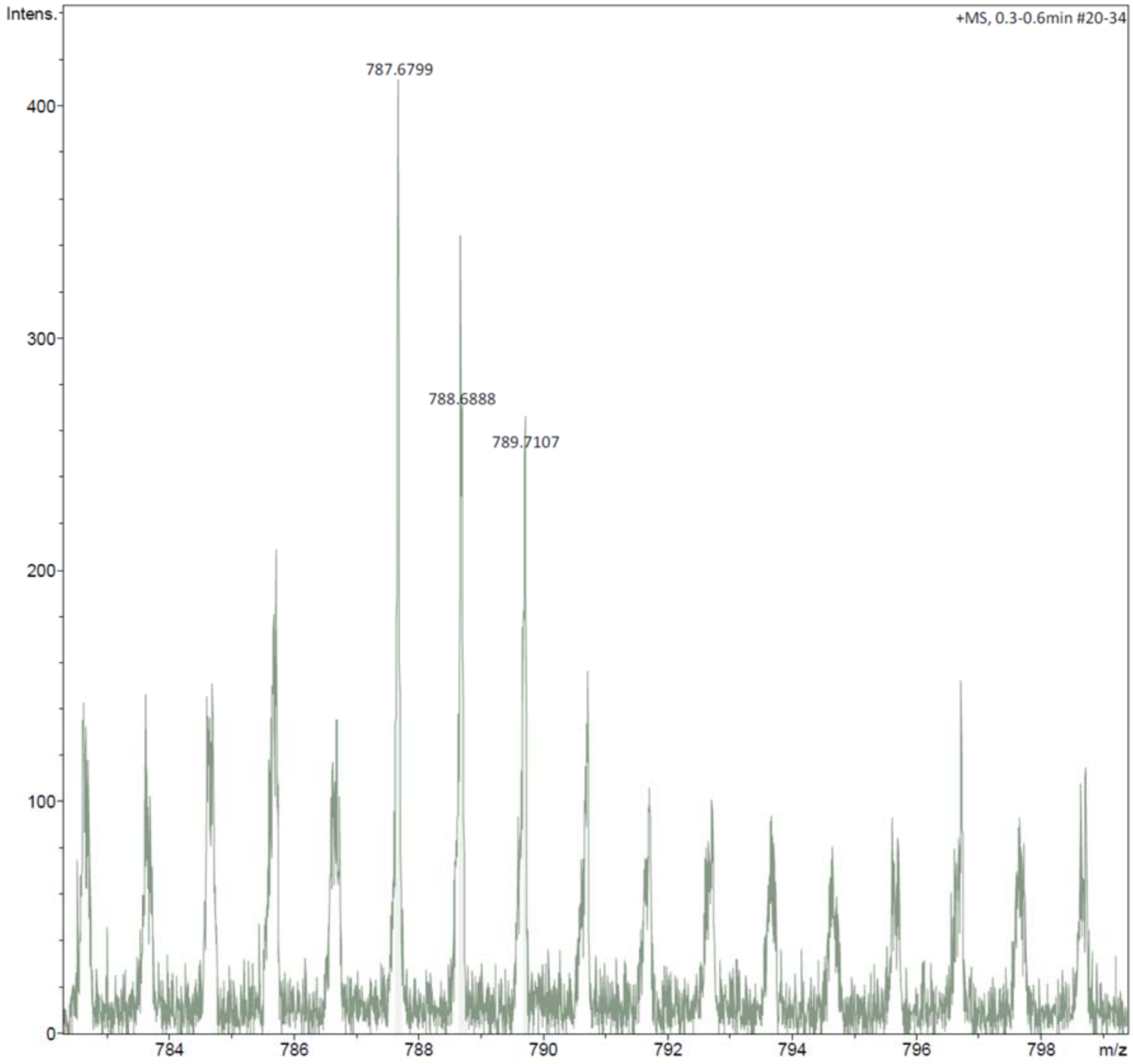

Figure S33. APCI mass spectrum of **Th7**.

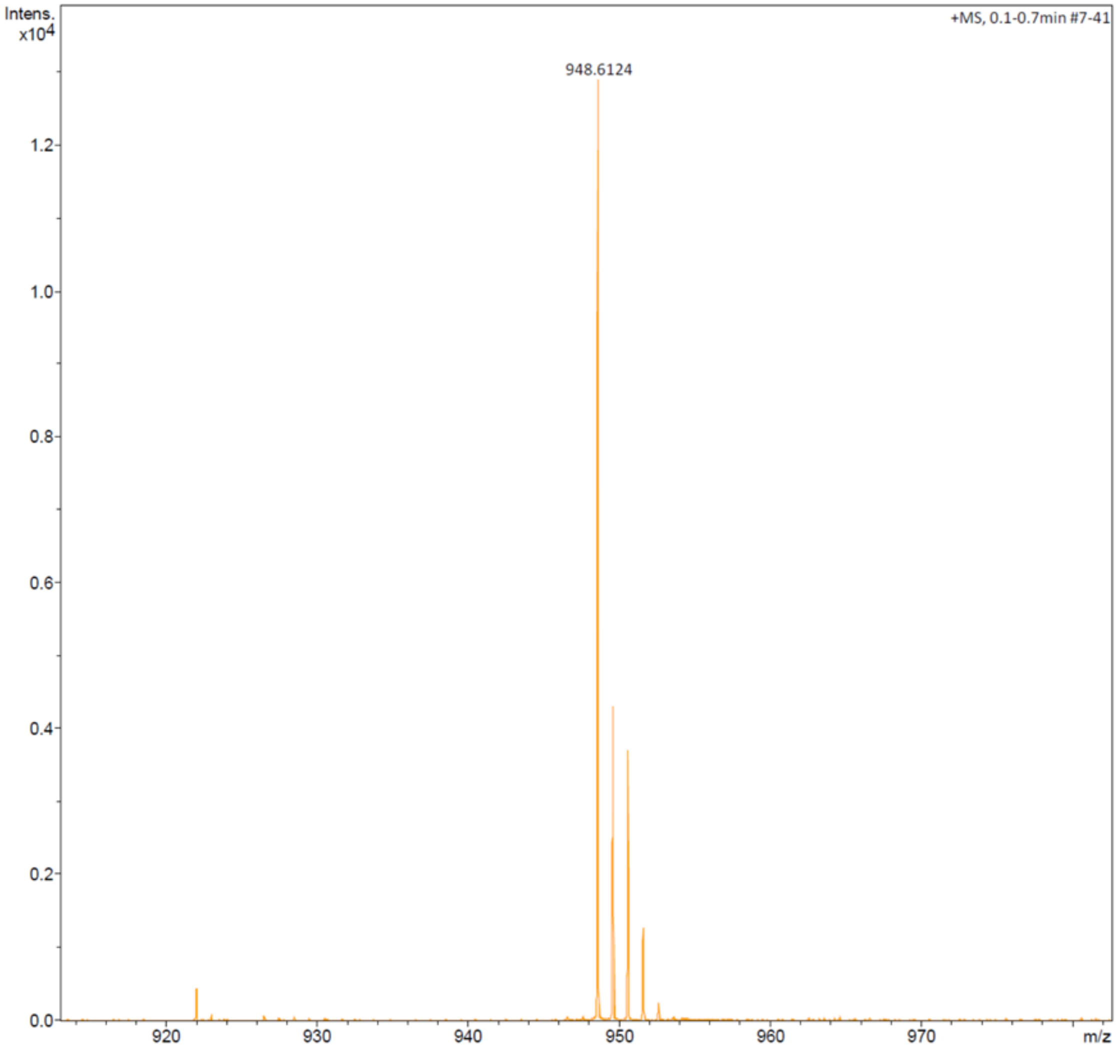

Figure S34. APCI mass spectrum of **Th8**.

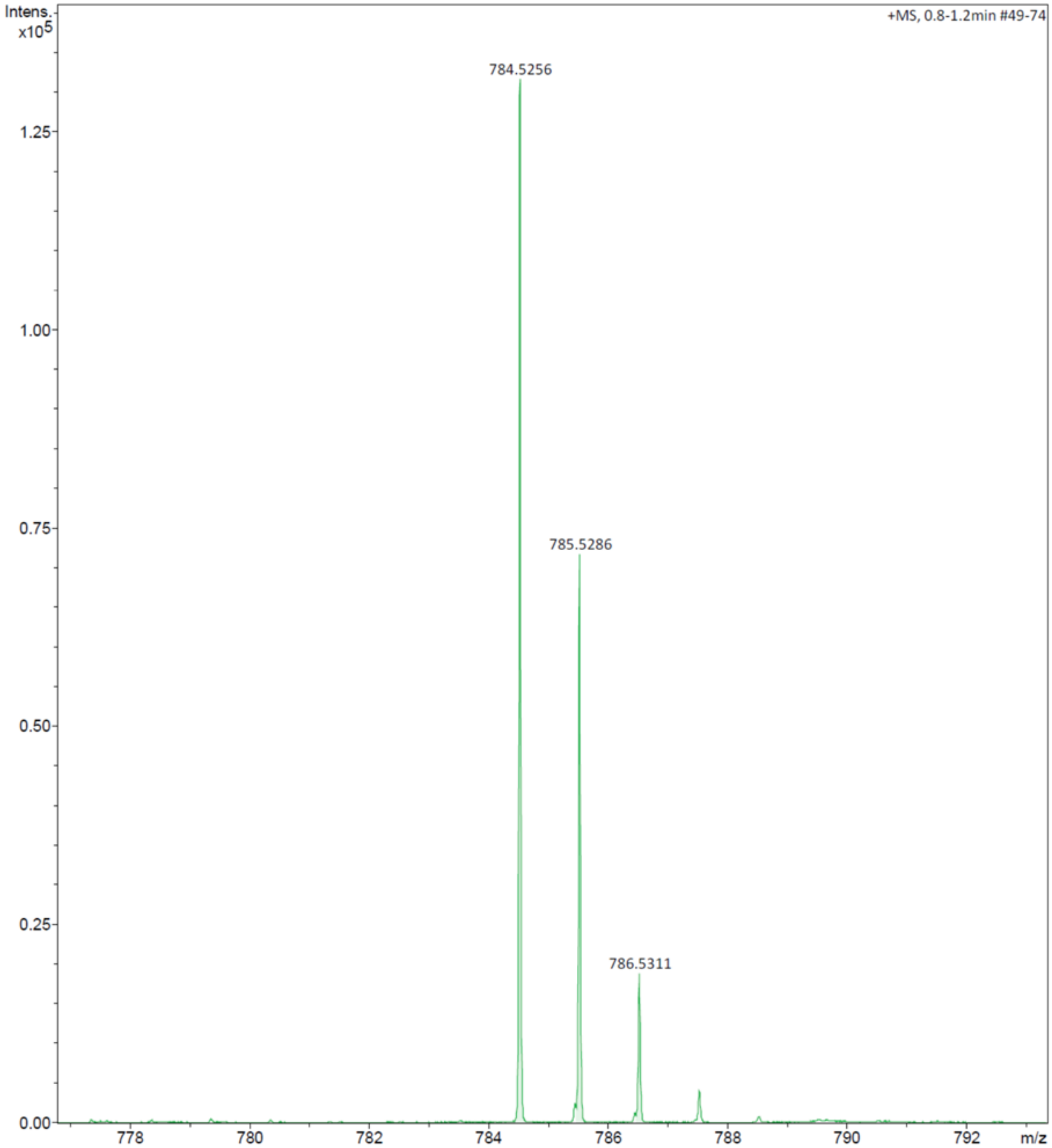

Figure S35. APCI mass spectrum of **Th9**.

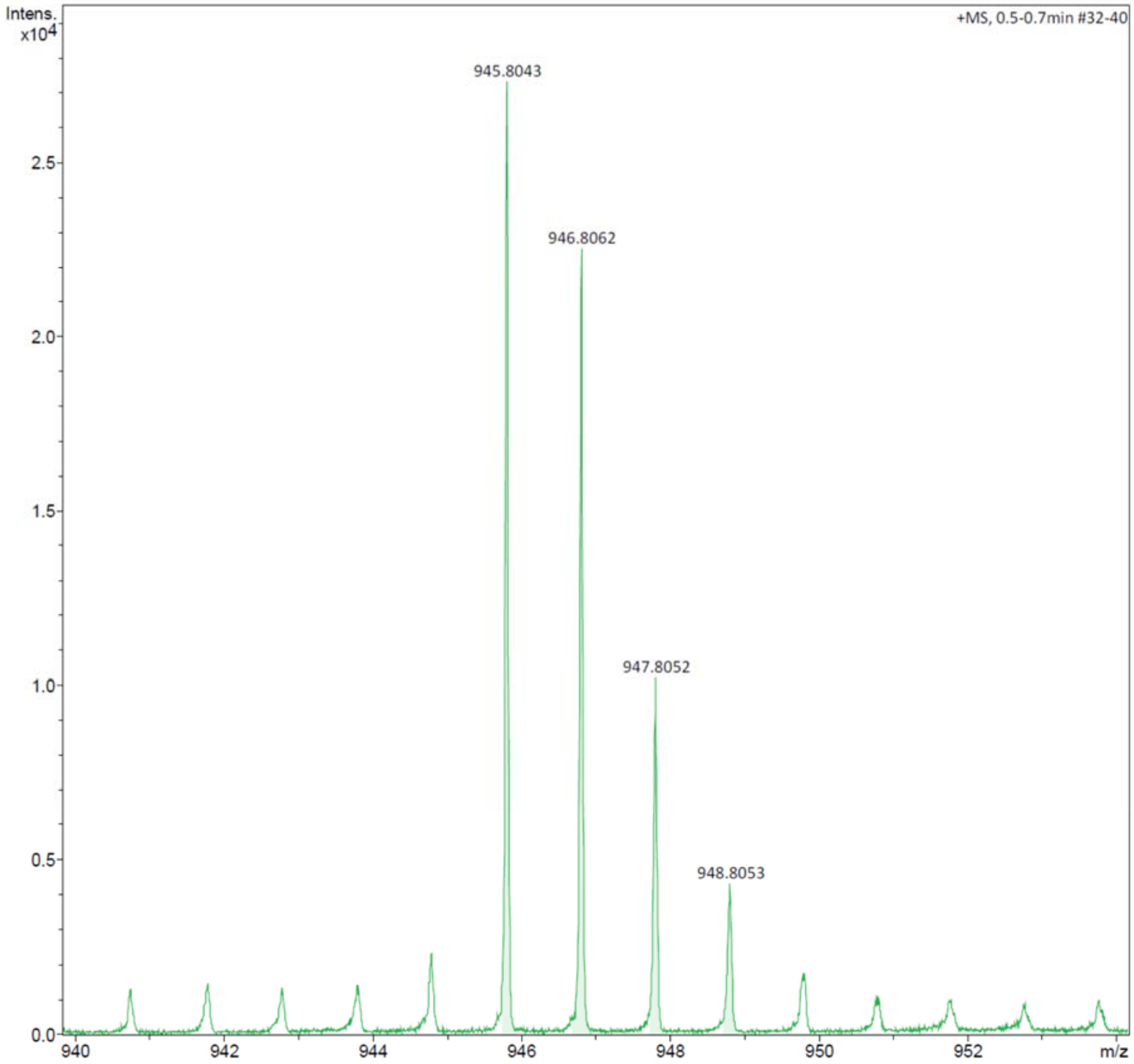

Figure S36. APCI mass spectrum of **Th10**.

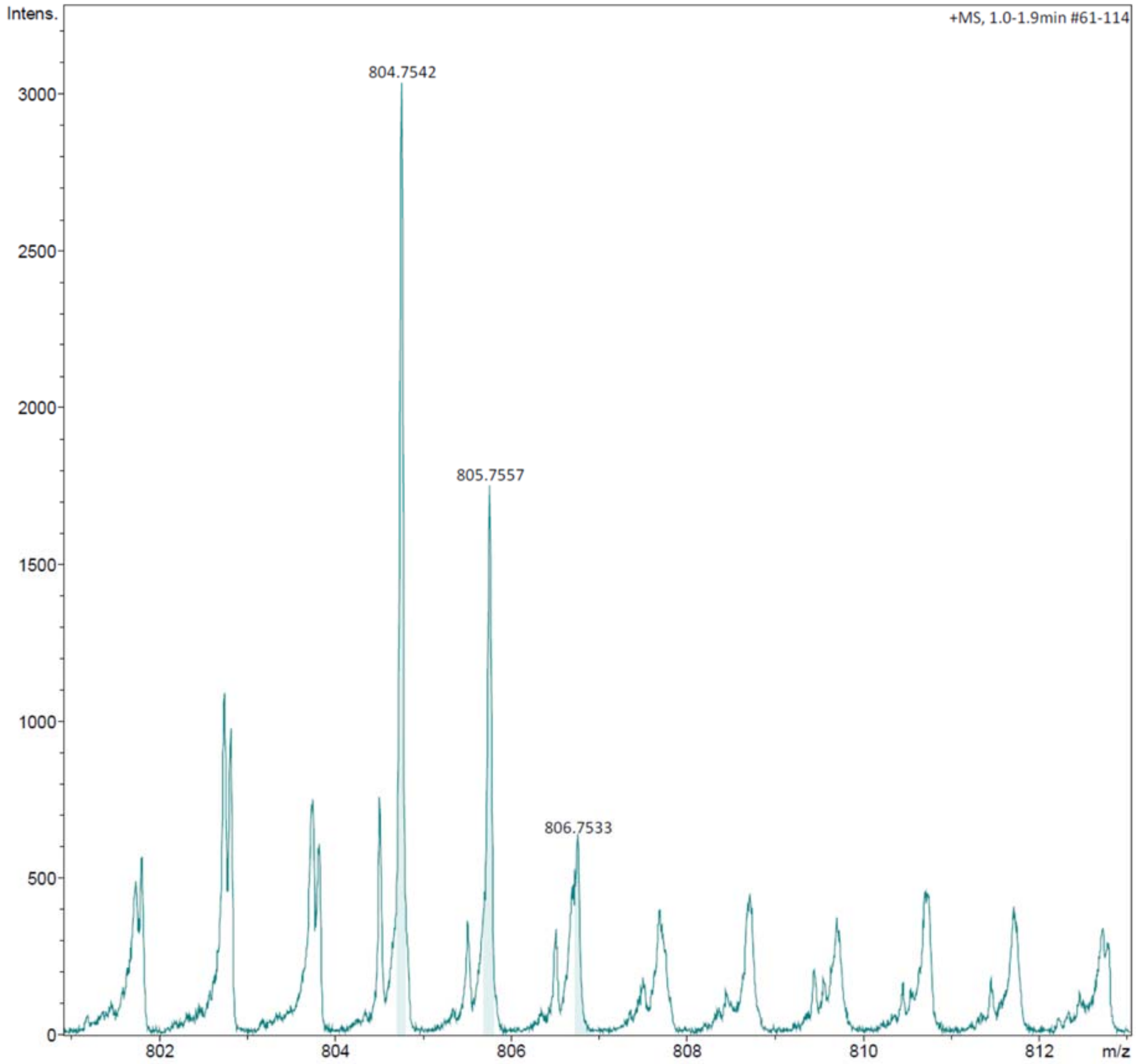

Figure S37. APCI mass spectrum of **Th11**.

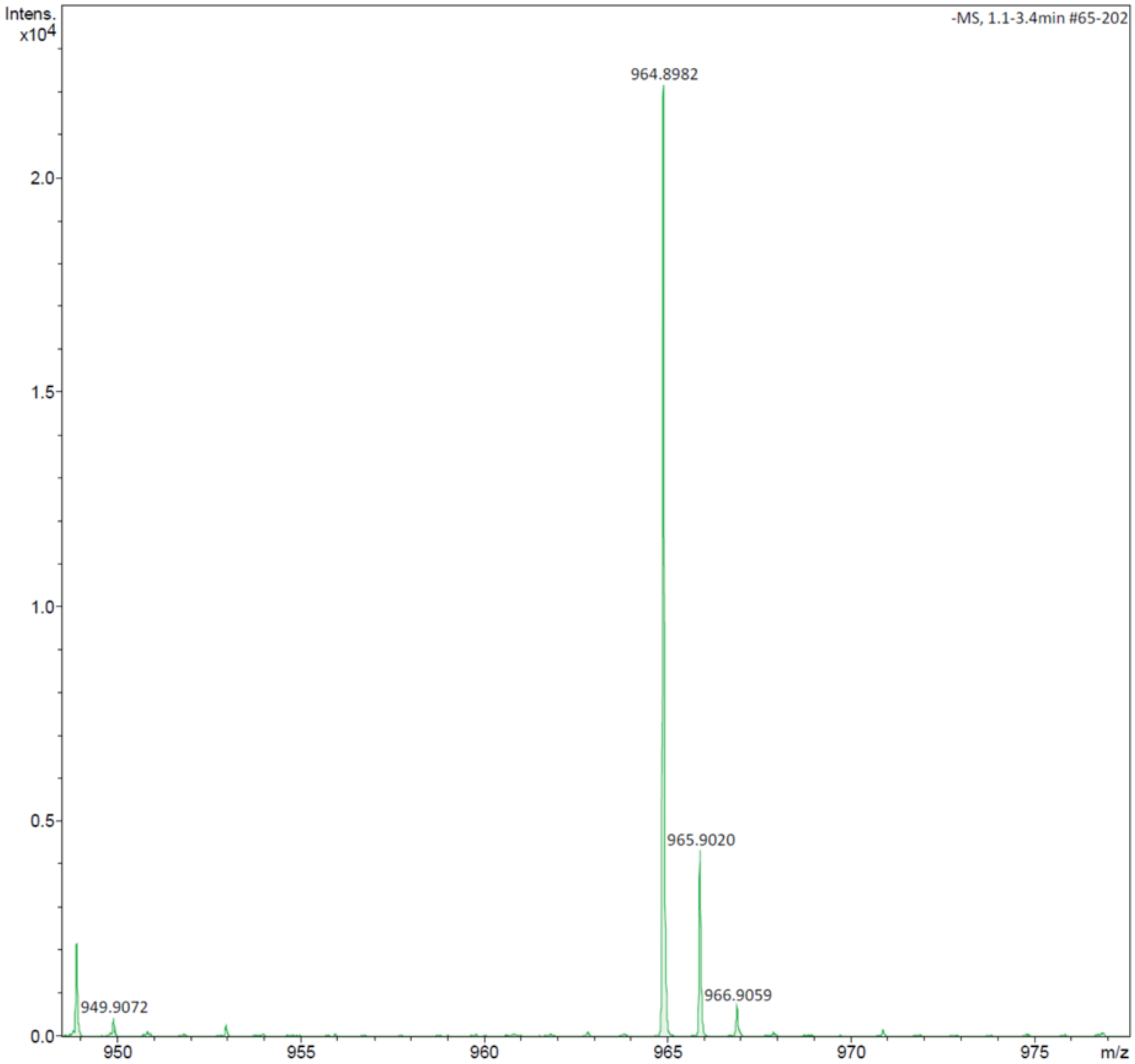

Figure S38. APCI mass spectrum of **Th12**.

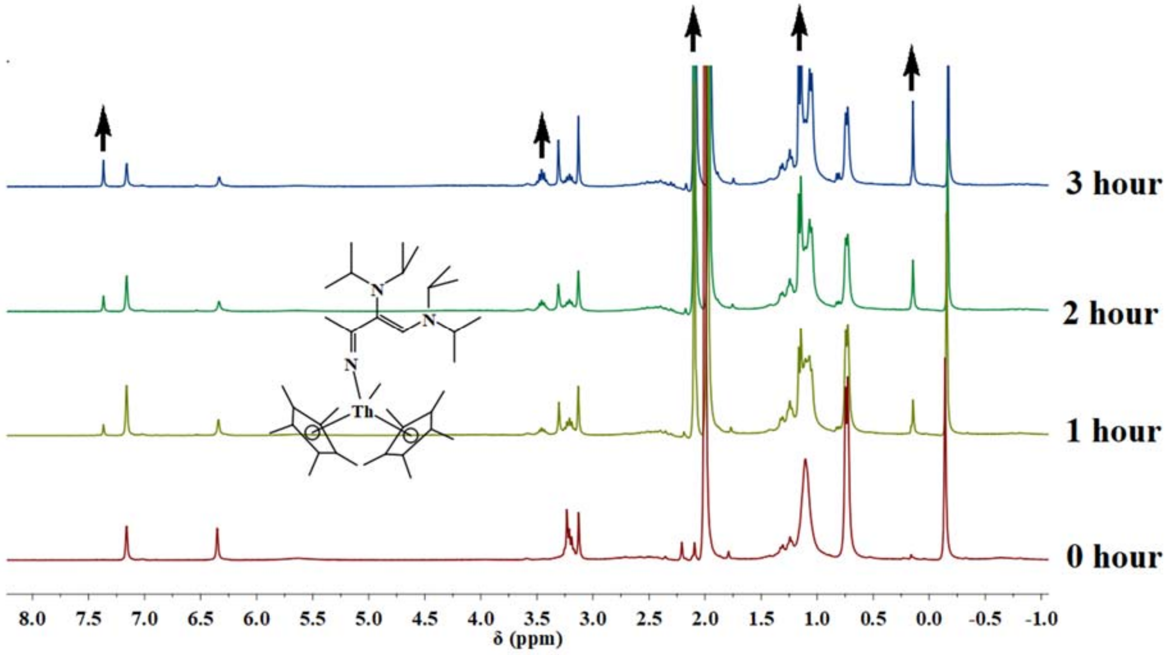

Figure S39. ^{1}H NMR analysis of reaction mixture of one equivalent **L1H** and two equivalent of $Cp^*{}_2Th(CH_3)_2$ in C_6D_6.

3. References

1. (a)Nacsa, E. D.; Lambert, T. H. Higher-Order Cyclopropenimine Superbases: Direct Neutral Brønsted Base Catalyzed Michael Reactions with α-Aryl Esters. *J. Am. Chem. Soc.* **2015**, *137*, 10246–10253. (b) Bruns, H.; Patil, M.; Carreras, J.; Vázquez, A.; Thiel, W.; Goddard, R.; Alcarazo, M. Synthesis and Coordination Properties of Nitrogen(I)-Based Ligands. *Angew. Chem. Int. Ed.* **2010**, *49*, 3680–3683.
